# Random mutagenesis of a hyperthermophilic archaeon identified tRNA modifications associated with cellular hyperthermotolerance

**DOI:** 10.1093/nar/gky1313

**Published:** 2019-01-03

**Authors:** Izumi Orita, Ryohei Futatsuishi, Kyoko Adachi, Takayuki Ohira, Akira Kaneko, Keiichi Minowa, Miho Suzuki, Takeshi Tamura, Satoshi Nakamura, Tadayuki Imanaka, Tsutomu Suzuki, Toshiaki Fukui

**Affiliations:** 1 School of Life Science and Technology, Tokyo Institute of Technology, Midori-ku, Yokohama 226-8501, Japan; 2 Department of Chemistry and Biotechnology, Graduate School of Engineering, University of Tokyo, 7-3-1 Hongo, Bunkyo-ku, Tokyo 113-8656, Japan; 3 Research Organization of Science and Technology, Ritsumeikan University, 1-1-1 Noji-higashi, Kusatsu 525-8577, Japan

## Abstract

Random mutagenesis for the hyperthermophilic archaeon *Thermococcus kodakarensis* was established by the insertion of an artificial transposon designed to allow easy identification of the transposon-inserted locus. The phenotypic screening was applied for the isolation of thermosensitive mutants of *T. kodakarensis*, which resulted in the isolation of 16 mutants showing defective growth at the supraoptimal temperature 93°C. The high occurrence of the mutants suggested that the high thermotolerance of hyperthermophiles was achieved by a combination of diverse gene functions. The transposon insertion sites in two-thirds of the mutants were identified in a group of genes responsible for tRNA modifications including 7-formamidino-7-deaza-guanosine (archaeosine), *N*^1^-methyladenosine/*N*^1^-methylinosine, *N*^4^-acetylcytidine, and *N*^2^-dimethylguanosine/*N*^2^,*N*^2^-dimethylguanosine. LC–MS/MS analyses of tRNA nucleosides and fragments exhibited disappearance of the corresponding modifications in the mutants. The melting temperature of total tRNA fraction isolated from the mutants lacking archaeosine or *N*^1^-methyladenosine/*N*^1^-methylinosine decreased significantly, suggesting that the thermosensitive phenotype of these mutants was attributed to low stability of the hypomodified tRNAs. Genes for metabolism, transporters, and hypothetical proteins were also identified in the thermosensitive mutants. The present results demonstrated the usefulness of random mutagenesis for the studies on the hyperthermophile, as well as crucial roles of tRNA modifications in cellular thermotolerance.

## INTRODUCTION

The hyperthermotolerance/hyperthermophily of hyperthermophiles has attracted great interest since their discovery, as most biomolecules are unstable under such extreme environments. It has been proposed that proteins produced by hyperthermophiles are highly rigid and able to retain their functional structure at high temperatures (1). The expression of one of two group II chaperonins and small heat shock proteins were up-regulated at higher temperature, possibly to promote refolding of denatured proteins (2–4). Hyperthermophiles also protect DNA and RNA from thermal denaturation by unique strategies. Reverse gyrase, an enzyme that introduces positive supercoiling to DNA structure (5), is encoded in the genomes of hyperthermophiles but not in any other mesophile genomes (6,7); indeed, reverse gyrase is essential for a hyperthermophile to grow at supraoptimal temperature (8). Hyperthermophiles produce unusual long linear polyamines for stabilizing dsDNA and stem parts of RNA and branched polyamines for stabilizing stem-and-loop structures in RNA (9,10). Accumulation of compatible solutes is also a thermoprotection strategy in hyperthermophiles (11–13).

*Thermococcus kodakarensis* strain KOD1, a sulfur-reducing hyperthermophilic euryarchaeon (14,15), has been regarded as one of useful model organisms for the studies on hyperthermophiles, because practical genetic techniques such as targeted deletion/insertion of genes based on homologous recombination (16–18) as well as transformation with shuttle vectors (19) have been developed in this organism. Targeted genetic manipulation is a powerful tool for the investigation of *in vivo* gene functions. For example, the functions of several novel enzymes and regulators involved in carbon and energy metabolism (20–25), including unique pentose metabolic pathways (26–28) have been elucidated. DNA replication has also been well studied in *T. kodakarensis* as a simple model of complex eukaryotic systems (29–31). However, the role of genes encoding proteins with unknown functions or with functions different from those predicted based on the primary structure remain difficult to determine. Random mutagenesis followed by phenotypic screening and identification of the responsible genes have contributed greatly to the elucidation of various gene functions in diverse organisms. Nevertheless, this approach has not been well adopted for hyperthermophiles, except in the case of UV mutagenesis to obtain 5-fluoroorotic acid-resistant uracil auxotrophic mutants of (hyper)thermophilic archaea *Sulfolobus acidocaldarius* (32), *Pyrococcus abyssi* (33), and *T. kodakarensis* (17). Meanwhile, random mutagenesis based on homologous recombination of a transposon-inserted genomic DNA library, prepared by *in vitro* transposition using hyperactive transposases, was reported for mesophilic pathogens *Haemophilus influenzae* and *Streptococcus pneumoniae* to determine the set of essential genes (34). This strategy was recently applied for the extremely thermophilic bacterium *Thermus thermophilus* (35) and hyperthermophilic archaeon *P. furiosus* (36), although the phenotypic screening from the mutant library has not been performed yet.

This study established a technique for random mutagenesis of *T. kodakarensis* by the insertion of an artificial transposon designed for easy recovery of the regions flanking the transposon on the chromosome. Then, we applied the technique for isolation of thermosensitive mutants of *T. kodakarensis*, resulting in the identification of several genes of which contribution to cellular hyperthermotolerance and hyperthermophily has not been reported so far. Of particular interest, the results provide new insight into relationship between tRNA modifications and survival of hyperthermophiles at extreme temperatures.

## MATERIALS AND METHODS

### Microorganisms and culture conditions

*Escherichia coli* and *T. kodakarensis* strains and plasmids used in this study are listed in Supplementary Table S1. *Escheichia coli* strains were cultivated at 37°C in Lysogeny broth (LB) medium. Ampicillin and gentamycin were added at a final concentration of 100 μg/ml and 30 μg/ml, respectively, when needed. *Thermococcus kodakarensis* strains were grown anaerobically at 85°C or 93°C in ASW-YT medium composed of 0.8-fold concentration of artificial seawater (ASW) (17), 10 g/l yeast extract and 5.0 g/l tryptone with either 2.0 g/l elemental sulfur (S^0^) (ASW-YT-S^0^) or 5.0 g/l sodium pyruvate (ASW-YT-Pyr), or in MA-YT-Pyr medium composed of 3.04 g/l Marine Art SF-1 (Osaka Yakken, Osaka, Japan), 10 g/l yeast extract, 5 g/l tryptone and 5 g/l sodium pyruvate (37). Plate media were solidified with Gelrite (Wako Pure Chemical Industries, Osaka, Japan) (17). All the anaerobic manipulations were done within the COY anaerobic chamber (COY Lab Products, Grass Lake, MI, USA).

### Genetic engineering

DNA manipulation was carried out by standard procedures, and restriction endonucleases and DNA modification enzymes were purchased from Takara Bio (Otsu, Shiga, Japan) or Toyobo (Osaka, Japan), unless otherwise noted. PCR reactions for gene cloning were carried out with KOD-Plus-ver.2 DNA polymerase (Toyobo), and those for other purposes such as colony PCR were done with KOD FX DNA polymerase (Toyobo). Supplementary Table S2 lists the sequences of oligonucleotide primers used in this study.

### Construction of the artificial transposon

A DNA fragment of *pdaD* along with the upstream region was amplified from *T. kodakarensis* gDNA as a template with the primer pair tk0149-f/tk0149-r. The amplified DNA fragment was 5′-phosphorylated and was inserted into a transposon vector pMOD3 (Lucigen (formerly Epicentre), Middleton, WI, USA) at the HincII site. A gentamicin-resistant cassette, amplified from pJQ200 (38) with the primer pair GAT-f2/GAT-r2-Age was inserted into the plasmid at the SmaI site, resulting in construction of pMOD3-Gm-pdaD. The artificial transposon region was amplified from pMOD3-Gm-pdaD with the primer pair pMOD3-MCS-Fw/pMOD3-MCS-Rv. The transposon fragment was digested by PvuII to obtain 5′-phospholyrated ends, and then was used for *in vitro* transposition.

### Random mutagenesis of *T. kodakarensis*

*Thermococcus kodakarensis* gDNA (150 μg) was fragmented by sonication, treated with Mung Bean Nuclease (Promega, Madison, WI, USA) for blunting of the ends, and then was separated by agarose gel electrophoresis. The 2–3-kb fragments were recovered from the gel, and the fragments (200 ng) were treated with A-attachment mix (Toyobo) according to the manufacturer's instruction. The 3′-A-attached DNA was ligated with the 3′-T-overhang vector pMD20 (Takara Bio) using T4 DNA ligase, and the reaction mixture was used to transform *E. coli* DH5α. The colonies of the transformants formed on plate media were suspended with fresh LB medium, and the library DNA was extracted from the cell suspension.

*In vitro* transposition of the artificial transposon into the library DNA was conducted using EZ-TN5 transposase (Lucigen) at 37°C for 2 h. The reaction mixture was used directly to transform *E. coli* HST08 Premium Competent Cells (Takara Bio) by electroporation. All of the colonies grown on the LB plate medium containing ampicillin and gentamycin were suspended within fresh LB medium, and the transposon-inserted library DNA was extracted from the suspended cells.

Random mutagenesis of *T. kodakarensis* was carried out by transforming *pdaD*-deleting agmatine-auxotrophic strains Δ*pdaD* or Δ2239 (see Supplementary Materials and Methods) with the transposon-inserted library DNA as follows. The cells grown in ASW-YT-S^0^ supplemented with 25 mg/l agmatine at 85°C were harvested from 3 ml culture, were resuspended within 200 μl of ASW and were kept on ice for 30 min. The transposon-inserted library DNA (typically 3 μg) was added to the suspension and was incubated on ice for 1 h. After outgrowth in ASW-YT-S^0^ medium at 85°C for 12 h, the cells harboring the transposon were selected by cultivation on ASW-YT-S^0^ plate medium not containing agmatine at 85°C for 1 day.

Southern blot analysis was carried out with the *pdaD* region, amplified using the tk0149-N/tk0149-r primer pair, as a specific probe. The preparation of the probe and the visualisation of the signal was performed using digoxigenin-labelled non-RI system (Roche Applied Sciences, Mannheim, Germany) according to the manufacturer's instructions.

### Screening of thermosensitive mutants

The single colonies in the random mutant library were picked and were inoculated into 200 μl of MA-YT-Pyr in each well of 96-well flat bottom plates. The plates were sealed with polyethylene terephthalate (PET) film (Watson Bio Lab, Tokyo, Japan) and were cultivated anaerobically at 85°C for 24 h. The cultivated plates were replicated to two plates by using the Bel-Blotter 96-well Replicating Tool (Bel-Art Products, South Wayne, NJ, USA) and were sealed with the PET film. The original plates were preserved anaerobically at 4°C. One of the replicated plate was cultivated at 85°C for 24 h, while the other was cultivated at 93°C for 24 h, anaerobically. A microplate reader (SUNRISE Rainbow RC-R, TECAN, Männedorf, Switzerland) was used to measure the optical density at 600 nm (OD_600_). Clones showing poor growth at 93°C but normal growth at 85°C comparable to the parent strain were selected as candidate mutants. The candidates in the original plate were isolated from single colonies using ASW-YT-S^0^ plate medium, and then were subjected to cultivation in 8 ml of MA-YT-Pyr liquid medium using a test tube with a butyl rubber stopper and screw cap as the second screening. The growth properties at 85°C and 93°C were determined by measuring OD_600_ of the culture in the tube directly with an S1200 diode array spectrophotometer (Biochrom, Berlin, Germany) at appropriate intervals and, thus, thermosensitive mutants were selected.

### Identification of transposon-inserted loci in *T. kodakarensis* random mutants

gDNA (3 μg) of the mutant strain was digested by XhoI or SalI, followed by self-ligation. The ligation mixture was used directly to transform *E. coli* EC100D *pir*-116 (Lucigen), and the rescued plasmid was obtained from the resulting transformants. Alternatively, inverse PCR using primers EzTN5-SqFP/EzTN5-SqRP was done to amplify the regions flanking the transposon. The nucleotide sequences of the plasmid and inverse PCR product were determined using pMOD-SqFP or pMOD-SqRP primers annealed within the transposon region to identify the transposon-flanking regions.

**Figure 1.**
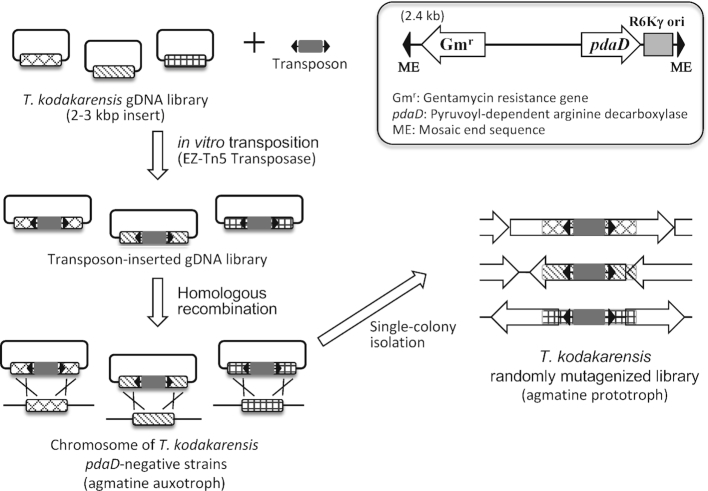
Overview of the strategy for construction of *T. kodakarensis* randomly mutagenized library based on *in vitro* transposition and double crossover homologous recombination.

### Complementation analysis

A plasmid pLCS was constructed for complementation analysis based on an *E. coli*–*T. kodakarensis* shuttle vector pLC71 (19) by inverse PCR with the primer set pLC71-inv1DKanr/pLC71-inv2 and successive self-ligation, by which *trpE* was replaced with multi-cloning sites downstream of the *pyrF* promoter. An intact coding region of the gene to be complemented was amplified from gDNA of *T. kodakarensis* KUW1 with the specific primers having appropriate restriction sites. The amplified fragment was digested at the sites, and then was inserted into pLCS at the corresponding sites. *T. kodakarensis* KD2239 was transformed by the resulting complementation vector, and the transformants were selected on ASW-YT-S^0^ plate medium supplemented with 30 μM lovastatin (16). The growth of the complemented strain was determined in an MA-YT-Pyr medium by test tube cultivation as described above.

### Preparation of total tRNAs from *T. kodakarensis*

Total RNA of *T. kodakarensis* was extracted from late-log phase cells grown on MA-YT-Pyr at 85°C. The corrected cells were resuspended in 10 volumes of solution D [4 M guanidine thiocyanate, 25 mM citrate–NaOH (pH 7.0) and 0.5% (w/v) *N*-lauroylsarcosine sodium salt], and then 8 volumes of neutralized phenol, 1 volume of 3 M sodium acetate (pH 5.3) and 1/15 volumes of 2-mercaptoethanol were added, followed by shaking for 1 h on ice. The cell suspension was mixed with 2 volumes of chloroform and was centrifuged at 10 000 *g* for 15 min at 4°C. The supernatant was collected and was mixed with 8 volumes of chloroform, followed by centrifugation at 10 000 *g* for 15 min at 4°C. The resultant supernatant was subjected to isopropanol precipitation. The pellet was dissolved in 10 volumes of 20 mM HEPES–KOH buffer (pH 7.5) containing 125 mM NaCl and was mixed with 4 volumes of 2-butoxyethanol. The mixture was placed on ice for 15 min and centrifuged at 10 000 *g* for 15 min at 4°C. Then, the recovered supernatants were subjected to ethanol precipitation. The obtained total RNAs were separated on 10% denaturing PAGE containing 7 M urea. The RNA bands were visualized by staining with 0.05% toluidine blue (Wako Pure Chemical Industries), and the bands corresponding to tRNAs were cut out from the gel. tRNAs were eluted from the gel using elution buffer [1 mM EDTA–NaOH (pH 8.0), 0.4 M sodium acetate (pH 5.3) and 0.1% SDS] and filtered with centrifugal filter units (Ultrafree-MC, HV, 0.45 μm, Merck Millipore). The eluted tRNAs were purified by ethanol precipitation and were desalted by drop-dialysis on a nitrocellulose membrane (MF-Millipore, Merck Millipore) against Milli-Q H_2_O for 2 h.

### Mass spectrometry analysis of nucleosides

Ten picomoles of tRNA mixtures were digested in 50 mM trimethylamine–acetate buffer (pH 5.3) containing 0.07 U nuclease P1 (Wako Pure Chemical Industries) and 0.08 U bacterial alkaline phosphatase (BAP.C75, Takara Bio) at 50°C for 2 h. The digests were dried *in vacuo* and were then dissolved in 20 μl of 90% acetonitrile, followed by LC/MS analysis as described previously (39,40) with some modification as follows. The samples were injected into a ZIC-cHILIC column (3 μm particle size, 2.1 × 150 mm, Merck Millipore) and eluted by 5 mM ammonium acetate (pH 5.3) (solvent A) and acetonitrile (solvent B) at a flow rate of 100 μl/min with a multistep linear gradient: 90–50% B from 0 to 30 min, 50% B for 10 min, 50–90% B from 40 to 45 min and, then, initialized to 90% B. Shotgun analyses were carried out as described previously (41) with slight modifications: Two picomoles of total tRNAs extracted from *T. kodakarensis* KD2239 and the mutants were digested at 37°C for 30 min in a reaction mixture (10 μl) composed of 20 mM NH_4_OAc (pH 5.3) and 10 or 50 units RNase T_1_ (Lucigen), or 20 mM NH_4_OAc (pH 7.7) and 10 ng RNase A (Ambion-Thermo Fisher Scientific, Waltham, MA, USA) and then subjected to capillary LC coupled to nano ESI/MS as described previously (39).

### Melting temperature measurement of tRNA mixtures

Twenty-five picomoles of tRNA mixtures were dissolved in degassed 20 mM HEPES–KOH (pH 7.5) buffer containing 100 mM NaCl and 1 mM MgCl_2_ and were incubated at 80°C for 5 min, followed by cooling at room temperature for annealing. A UV-Vis spectrophotometer (V-630, JASCO) equipped with an 8 multi-quartz micro cell array (path length: 10 mm, JASCO) was used to monitor the hyperchromicity. The temperature gradient was as follows: 25°C for 30 sec, ramped at 5°C/min to 40°C and held for 5 min and after that ramped at 0.5°C/min to 105°C. The measurement was performed in triplicate for each sample.

## RESULTS

### Random mutagenesis of *T. kodakarensis*

We initially tried to use an active transposon isolated from the crenarchaeon *Sulfolobus solfataricus* (42) for random mutagenesis in *T. kodakarensis*; however, no evidence of transposition could be obtained. The next examination was based on random insertion into a gDNA plasmid library (average gDNA length was ∼0.8 kb) by single-crossover homologous recombination. Although the circular DNA was actually inserted into the chromosome, the transformation efficiency was too low (∼10^1^/μg DNA) for practical applications.

**Figure 2.**
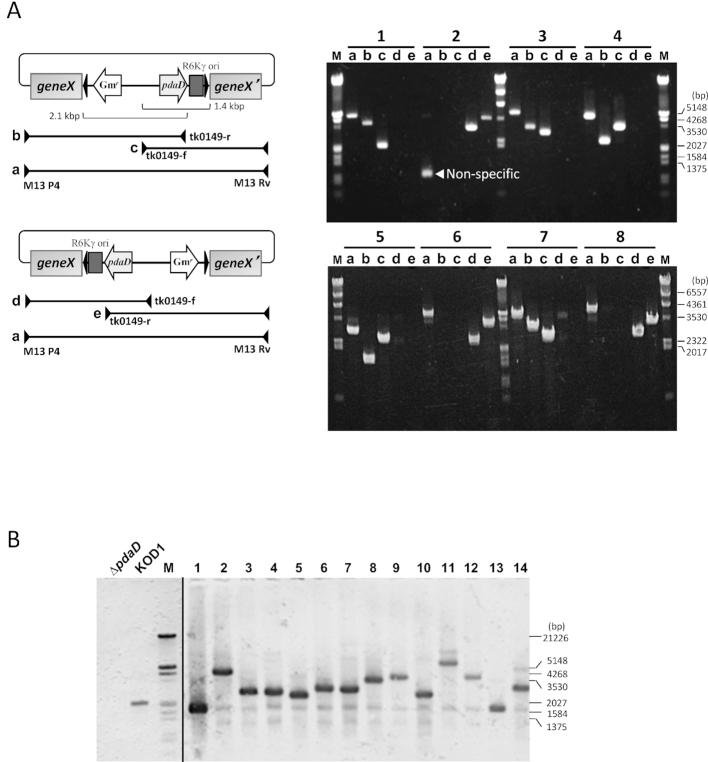
A PCR analysis of the transposon-inserted gDNA library. The plasmid extracted from randomly selected eight *E. coli* clones in the library was used as a template. PCR was carried out with a primer pair of (a) M13P4/M13Rv, (b) M13P4/tk0149-r, (c) tk0149-f/M13Rv, (d) M13P4/tk0149-f and (e) tk0149-r/M13Rv. (**A**) The primer annealing sites are shown by arrowheads. (**B**) Southern blot analysis of *T. kodakarensis* transposon-inserted clones with the *pdaD* probe. Lanes: Δ*pdaD*, genomic DNA from the strain Δ*pdaD*; KOD1, genomic DNA from the wild strain KOD1; 1–14, genomic DNA from randomly selected 14 clones in the transposon-inserted mutant library of *T. kodakarensis*; M, molecular size marker.

Further efforts were made to achieve random insertion of a *pdaD* marker gene into the chromosome of a *pdaD*-lacking, agmatine-auxotrophic mutant of *T. kodakarensis* by double-crossover homologous recombination. For this purpose, *in vitro* transposition of an artificial transposon was adopted to construct a gDNA library in which both the ends of the marker gene were flanked by gDNA regions. Namely, a Tn5-based artificial transposon containing a *pdaD* marker, gentamicin resistance cassette and R6Kγ ori (Figure 1) was subjected to *in vitro* transposition into the *T. kodakarensis* gDNA library (2–3 kb fragments, 3 × 10^4^ clones) using EZ-Tn5 transposase (43). Following the transposase treatment, the gDNA library was then introduced into *E. coli* by electroporation, resulting in construction of a transposon-inserted gDNA library (1 × 10^5^ clones). PCR analysis of 30 randomly selected clones indicated that 25 clones had the transposon within the gDNA region, among which 11 clones possessed gDNA regions longer than 0.5 kb, possibly enough for homologous recombination in *T. kodakarensis*, flanked by both ends of the transposon. Figure 2A shows the results of the PCR analysis for eight selected clones. Approximately 4 × 10^4^ clones were estimated to be capable of inducing double-crossover homologous recombination in *T. kodakarensis*. It was roughly estimated that the DNA library clones capable of transposon insertion covered the *T. kodakarensis* genome with ∼13-fold redundancy, which was expected to be sufficient for genome-wide insertional mutagenesis.

*T. kodakarensis* strain Δ*pdaD* was then transformed with the transposon-inserted library DNA. The consequent double-crossover homologous recombination events allowed us to obtain transformants having the transposon insertion into the chromosome with an efficiency of ∼10^3^/μg DNA. Southern blot analysis of 14 transformants with the *pdaD* probe indicated that all the examined clones harbored one transposon region in the chromosome at a random locus (Figure 2B). We confirmed further that the transposon-inserted region could be rescued in the *E. coli pir^+^* strain by transforming with gDNA of the mutants after XbaI- or SalI-digestion and successive self-ligation (Supplementary Figure S1A). The validation of this random mutagenesis approach was demonstrated by isolation of 6-methylpurine-resistant mutants (see Supplementary Results and Figure S1B and C).

### Screening of thermosensitive mutants using 96-well plates

The randomly mutagenized library of *T. kodakarensis* was constructed using Δ2239 as a host strain, since Δ2239 showed higher transformation efficiency than Δ*pdaD* due to lack of a gene encoding a putative type II restriction endonuclease (see Supplementary Results). Then, the mutant library was used for the isolation of mutants sensitive to the supraoptimal temperature 93°C. The cultivation was carried out using 96-well plates as described in Materials and Methods. We screened 1957 clones in the randomly mutagenized library and obtained 45 clones showing lower OD_600_ at 93°C but similar OD_600_ at 85°C when compared to the control strain. The second screening in 8 ml of MA-YT-Pyr medium resulted in the isolation of 16 mutant clones showing growth deficiency at 93°C, while 9 candidates were false-positive clones with comparable growth at 93°C to that at 85°C. Twenty other candidates were classified as partially thermosensitive mutants because they exhibited slower or poorer growth (OD_600_ < 1) at 93°C than the control strain.

### Identification of the transposon-inserted loci

The transposon-inserted region in the isolated mutants was rescued in the *E. coli pir*^+^ strain and was identified by nucleotide sequencing of the regions flanking the transposon (Supplementary Figure S1A). Since the plasmid rescue was unsuccessful for unknown reasons, the region in the two partially thermosensitive mutants FFH03 and FFH19 were identified by sequencing the inverse PCR products. The identified mutation loci in the 16 strictly thermosensitive mutants and 20 partially thermosensitive mutants are listed in Table 1 and Supplementary Table S3, respectively.

**Table 1.** Transposon insertion sites in *T. kodakarensis* thermosensitive mutants isolated by random mutagenesis

Predicted function	Transposon insertion site	Annotation	Mutant strain (direct repeat region [bp no.])	Complementation
DNA topology	*tk0470*	Reverse gyrase	FFH11 (1891–1899)	Not tested (8)
tRNA modification	*tk0754*	tRNA (Met) cytidine acetyltransferase (TmcA)	FFH02, FFH32 (1522–1530)	+
	*tk0760*	7-Cyano-7-deazaguanine tRNA-ribosyltransferase (TgtA)	FFH16, FFH17, FFH18 (962–970), FFH21 (1167–1175)	+
	*tk0981*	Guanine10-*N*^2^-dimethyltransferase (Trm11)	FFH35 (105–113)	+
	*tk1198*	Organic radical activating protein (QueE)	FFH05 (595–603), FFH24 (497–505)	+
	*tk1328*	*N* ^1^-methyladenosine methyltransferase (TrmI)	FFH20 (135–143)	+
metabolism	*tk0672*	Putative glutamate synthase β-subunit	FFH15 (283–291)	-
	*tk1402*	Polysaccharide deacetylase	FFH08 (526–534)	-
transport	*tk1803*	Dipeptide/oligopeptide ABC transporter permease	FFH12 (141–149)	+
unknown	*tk0647*	Hypothetical protein	FFH27 (582–590)	-
	*tk2145*	Hypothetical protein	FFH22 (518–526)	+

**Figure 3.**
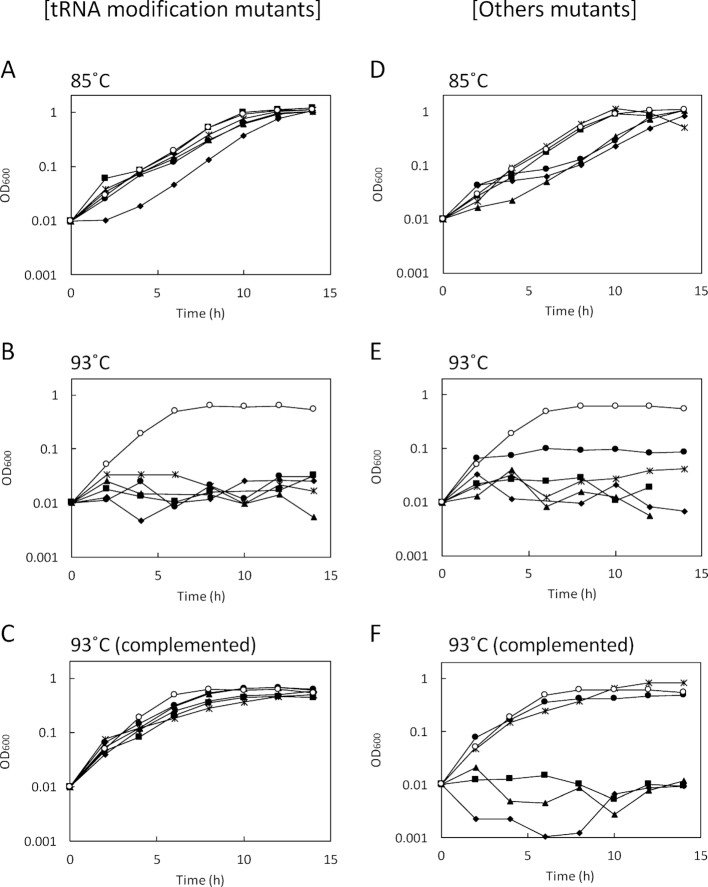
Growth properties of *T. kodakarensis* thermosensitive mutants (left) and the complemented strains (right) isolated from the transposon (Tn)-inserted random mutant library. The cells were cultivated in 8 ml MA-YT-Pyr medium in a test tube (*n* = 3). Left: Growth of tRNA modification mutants at 85°C (**A**) and 93°C (**B**), and growth of the complemented strains at 93°C (**C**). (○) KD2239 control strain; (✦) FFH05 (*queE*::Tn); (▴) FFH18 (*tgtA*::Tn); (•); FFH20 (*trmI*::Tn); (▪)FFH32 (*tmcA*::Tn); (×) FFH35 (*trm11*::Tn). Right: Growth of other mutants at 85°C (**D**) and 93°C (**E**), and growth of the complemented strains at 93°C (**F**). (○) KD2239 control strain; (✦) FFH15 (*tk0672*::Tn); (▴) FFH08 (*tk1402*::Tn); (•) FFH12 (*tk1803*::Tn); (▪) FFH27 (*tk0647*::Tn); (×) FFH22 (*tk2145*::Tn). In the panels (C) and (F), growth of the strains transformed with the corresponding complementation vector are shown, except for the control strain KD2239.

We then focused on the 16 mutants showing strict thermosensitivity. One mutant FFH11 was determined to have the transposon within the reverse gyrase gene (*tk0470*). Interestingly, the transposon insertion was identified within predicted tRNA modification genes for 10 mutants. Two and four mutants had the transposon insertion within *tk1198* and *tk0760*, annotated as *queE* and *tgtA*, encoding organic radical activating protein and 7-cyano-7-deazaguanine tRNA-ribosyltransferase, respectively. Both of the genes have been proposed to function in archaeosine (7-deaza-guanosine derivative [G^+^]) biosynthesis. Two different sites of transposon insertion were observed for each gene (Table 1). Archaeosine is seen at position 15 in the D-loop of archaeal tRNAs, and QueE catalyzes the third step in the synthesis of 7-cyano-7-deazaguanine (preQ_0_), which is a precursor base of archaeosine. TgtA is a tRNA-guanine transglycosylase that replaces guanine base at position 15 of tRNA by preQ_0_ to form G^+^ (44–47). The mutation loci in the 4 mutants were identified to be *tk0754* (2 mutants had the transposon insertion at the same site), *tk0981* and *tk1328*. TK0754 shared 20% identity with tRNA(Met) cytidine acetyltransferase (TmcA) from *E. coli*. TmcA is a GNAT family acetyltransferase forming *N*^4^-acetylcytidine derivative (ac^4^C) at the wobble position of elongator tRNA^Met^ in *E. coli* (48). TK0981 is an archaeal tRNA guanine10-*N*^2^-dimethyltransferase (aTrm11), a kind of THUMP domain-containing, *S*-adenosylmethionine (SAM)-dependent methyltransferase. aTrm11 from the closely related hyperthermophile *P. abyssi* catalyzed mono-and di-methylation of *N*^2^-amino group of guanine at position 10 to produce *N*^2^-methylguanosine (m^2^G10) and *N*^2^,*N*^2^-dimethylguanosine (m^2,2^G10), unlike the ortholog from yeast *Saccharomyces cerevisiae*, which only catalyzed mono-methylation (49,50). TK1328 is an ortholog of the SAM-dependent tRNA adenine58-*N*^1^-methyltransferase (TrmI), which forms *N*^1^-methyladenosine (m^1^A) at position 58 in the T-loop of many tRNAs. The significant difference between archaeal TrmI (aTrmI) and the bacterial and eukaryotic orthologs was the ability to modify not only A58 but also A57, and the resulting m^1^A57 was thought to be a precursor of 1-methylinosine (m^1^I57) (51–53). The growth properties of the five kinds of predicted RNA modification mutants at 85°C and 93°C are shown in Figure 3A and B, respectively, and individually shown in Supplementary Figure S2. The mutants exhibited comparable growth with the control strain KD2239 except for FFH05 (one of *queE*::Tn mutants) showing slightly longer lag phase before the growth at 85°C, while they all did not grow at 93°C.

The transposon insertions in other isolates were identified within *tk0672, tk1402, tk1803*,*tk0647* and *tk2145*. TK0672, TK1402 and TK1803 have been annotated as putative glutamate synthase β-subunit, polysaccharide deacetylase and dipeptide/oligopeptide ABC transporter permease, respectively. TK0647 and TK2145 are hypothetical proteins with unknown function. FFH08 (*tk1402*::Tn) had a longer lag phase, and FFH15 (*tk0672*::Tn) and FFH12 (*tk1803*::Tn) showed slightly slower growth at 85°C, although the final OD_600_ values of these strains reached the same level as that of the control strain (Figure 3D). Two mutants of hypothetical protein FFH27 (*tk0647*::Tn) and FFH22 (*tk2145*::Tn) showed almost the same growth properties as the control strain at the optimum temperature (Figure 3D). At the higher temperature 93°C, FFH12 poorly grew to an OD_600_ of 0.1, and the other four mutants exhibited growth deficiency as shown in Figure 3E. The individual growth properties are also shown in Supplementary Figure S3.

### Complementation analysis

Complementation analysis was carried out by introducing the corresponding intact gene into the isolated mutants using a *E. coli*-*T. kodakarensis* shuttle vector pLCS, which was designed for constitutive gene expression with moderate strength in *T. kodakarensis* under the control of the *pyrF* promoter. Notably, the growth of the five types of the predicted tRNA modification mutants at 93°C was almost completely restored by genetic complementation, strongly suggesting that the tRNA modifications were associated with the ability to grow at higher temperatures. The mutants with transposon insertions in *tk1803* and *tk2145* were also complemented. No growth restoration at the higher temperature was observed for *tk0672, tk1402* and *tk0647* mutants (Figure 3C and F, and Supplementary Figures S2 and S3).

### Mass spectrometric analyses of tRNAs in the mutants associated with tRNA modifications

**Figure 4.**
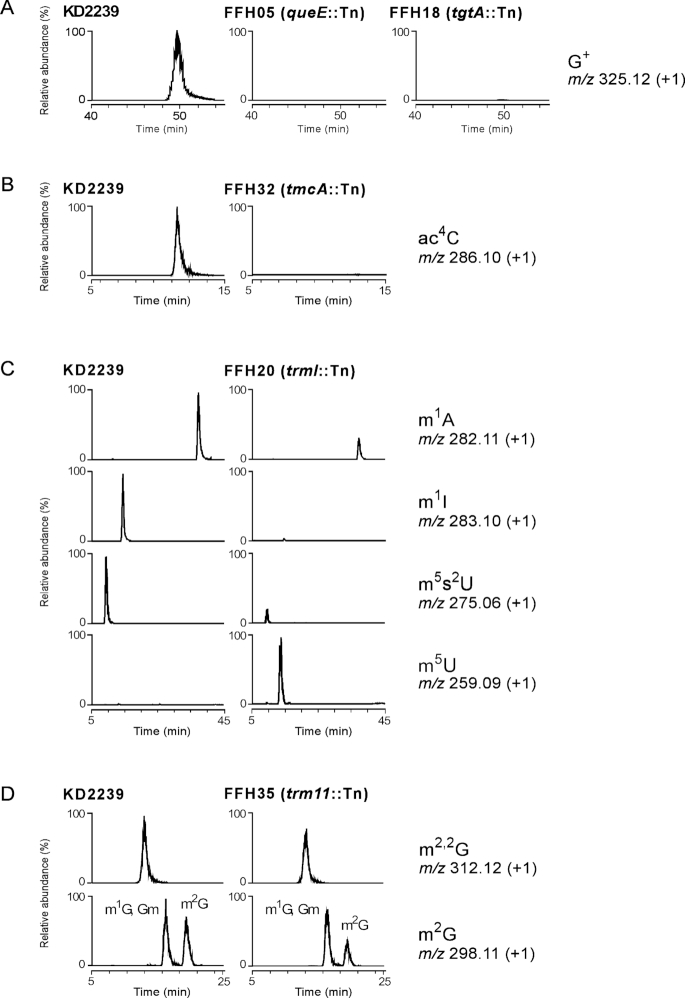
LC–MS/MS analysis of total tRNA nucleosides prepared from *T. kodakarensis* thermosensitive mutants having transposon insertion within a predicted tRNA-modification gene. The panels represent mass chromatograms that detect monovalent positive ions of the modified nucleosides of G^+^ (**A**), ac^4^C (**B**), m^1^A, m^1^I, m^5^s^2^U and m^5^U (**C**) and m^2,2^G and monomethyl guanosines (**D**), respectively. The vertical axes show relative abundance (%) of each the modified nucleoside normalized by 5-methylcytidine. The horizontal axes show retention time (min).

Total tRNA nucleosides prepared from the predicted tRNA-modification mutants were analyzed by LC–MS/MS, and the relative abundance of the modified nucleoside was compared with that from the control strain KD2239. In the mass chromatograms, G^+^ disappeared in both FFH05 (*queE*::Tn) and FFH18 (*tgtA*::Tn) (Figure 4A) and ac^4^C was absent in FFH32 (*tmcA*::Tn) (Figure 4B), demonstrating that these genes are responsible for the respective tRNA modifications. A shotgun analysis of tRNA fraction from KD2239 successfully detected 14 species of RNase T_1_-digested fragments containing G^+^ at position 15 (Supplementary Figure S4 and Supplementary Table S4A). Sequences of the 10 fragments were further proved by collision-induced dissociation (CID) analysis, and the presence of G^+^ at position 15 in each fragment was confirmed (Supplementary Figure S5). These fragments covered total species of tRNAs (Supplementary Table S4A), suggesting that G^+^15 is potentially present in all tRNAs in *T. kodakarensis*. All of the G^+^-containing fragments disappeared in FFH05 (Supplementary Figure S4).

In the tRNAs from FFH20 (*trmI*::Tn), m^1^A significantly decreased, and m^1^I completely disappeared (Figure 4C). It has been known that m^1^A is present at positions 9 and 58 and m^1^I is only present in position 57 (51, 54), and aTrmI introduces methylations at positions 57 and 58 (51–53). The shotgun analysis of tRNA fraction from KD2239 detected 4 species of RNA fragments bearing m^1^I57 and/or m^1^A58 covering total species of tRNAs, and no hypomethylated fragment was detected (Supplementary Figure S6 and Supplementary Table S4B). Sequences of the fragments with m^1^I/m^1^A from KD2239 and those without the modification(s) from FFH20 were confirmed by CID analysis (Supplementary Figure S7). These results clarified that m^1^A58 is a ubiquitous modification in all tRNAs, and m^1^I57 is also introduced along with m^1^A58 into all the corresponding tRNAs. Strikingly, we observed significant reduction of 5-methyl-2-thiouridine (m^5^s^2^U) and massive accumulation of 5-methyluridine (m^5^U) in FFH20, indicating that 2-thiouridylation of m^5^U to form m^5^s^2^U was down-regulated when m^1^I57/m^1^A58 were absent.

The relative abundances of m^2,2^G and m^2^G in tRNA from FFH35 (*trm11*::Tn) decreased slightly in the total nucleoside analysis (Figure 4D). This was probably due to the function of TK0970 of which *Pyrococcus* homolog (aTrm1) was reported to form m^2^G26 and m^2,2^G26 (56). The role of aTrm11 in the formation of m^2,2^G and m^2^G at position 10 of tRNAs was confirmed by the shotgun analysis of tRNA fraction from both KD2239 and FFH35. We detected two RNase T_1_-digested fragments containing m^2,2^G and m^2^G at position 10 from KD2239 (Supplementary Figure S8), and confirmed disappearance of them in tRNAs from FFH35. On the other hand, further two fragments estimated to bear m^2,2^G26 were not changed by the *trm11*::Tn mutation (Supplementary Figure S8). The position of each modification was confirmed by CID analysis (Supplementary Figure S9). The RNA fragments with m^2^G or m^2,2^G are predicted to be derived from 22 or 7 species of tRNAs (Supplementary Table S4C).

### Melting temperature of total tRNAs in the mutants

The effects of the respective modifications on thermal stability of tRNAs were estimated by measuring melting temperatures (*T*_m_) of total tRNAs prepared from the control strain KD2239 and the isolated mutants. The *T*_m_ value of total tRNA from KD2239 was determined to be 91.3°C (Table 2), and we observed significant reduction in *T*_m_ by 1.5°C∼2°C for tRNA fractions from FFH05 (*queE*::Tn), FFH18 (*tgtA*::Tn) and FFH20 (*trmI*::Tn). Considering the fact that G^+^15 is ubiquitously found in total tRNAs (Supplementary Figure S4), it is plausible that G^+^15 contributes to thermal stabilization of tRNAs in *T. kodakarensis*. The low *T*_m_ value of tRNAs from FFH20 can be explained by the hypomodification of m^5^s^2^U54 in addition to loss of m^1^I57/m^1^A58 (Figure 4C). In contrast, such the decrease in *T*_m_ was not observed for tRNAs from FFH32 (*tmcA*::Tn) and FFH35 (*trm11*::Tn) despite the cellular thermosensitivity.

**Table 2.** Melting temperature of total tRNA extracted from *T. kodakarensis* thermosensitive mutants having a transposon insertion within tRNA-modification genes

Strain	Relevant genotype	*T*_m_ of total tRNA (°C)	*P* value
KD2239		91.3 ± 0.3	
FFH05	*queE*::Tn	89.4 ± 0.2	0.0017**
FFH18	*tgtA*::Tn	89.9 ± 0.2	0.0044**
FFH20	*trmI*::Tn	89.6 ± 0.3	0.0034**
FFH32	*tmcA*::Tn	91.0 ± 0.1	0.14
FFH35	*trm11*::Tn	91.8 ± 0.4	0.26

*P*-values were calculated in comparison with control strain KD2239 using Student's *t*-test.

**Indicates *P*-value <0.005.

## DISCUSSION

The random mutagenesis of *T. kodakarensis* was achieved by homologous recombination of the transposon-inserted DNA, prepared by *in vitro* transposition of an artificial transposon into a genomic DNA library using the natural competency of *T. kodakarensis*. In the present system, the transposon was designed for the easy recovery of the transposon-inserted region by plasmid rescue into *E. coli*, and a strain lacking a putative restriction endonuclease gene (*tk2239*) was used as a host strain to increase transformation efficiency. This study further applied the mutagenesis system to phenotypic screening of *T. kodakarensis*. Here we focused on the thermosensitive phenotype to obtain new knowledge for the hyperthermotolerance/hyperthermophily, one of the most intriguing properties of hyperthermophiles.

The screening of 1957 mutant clones by 96-well plate cultivation resulted in isolation of 16 mutants showing growth deficiency at the supraoptimal temperature 93°C. Moreover, 20 additional mutants were isolated as partially thermosensitive mutants with slower or poorer growth at 93°C when compared to the control strain. It was surprising that the occurrence of the mutants reached 1.8% of the screened clones, which strongly suggested that the cellular tolerance to supraoptimal temperature was achieved by a combination of various gene functions. Notably, one isolate was identified to be a mutant of reverse gyrase (TK0470), which has been known to be essential for *T. kodakarensis* to grow at 93°C (8). The isolation of the reverse gyrase mutant demonstrated the validity of the present random mutant library for phenotypic screening.

It was of interest that two-thirds of the isolated mutants had transposon insertions within genes predicted to function in tRNA modifications. Post-transcriptional modifications of tRNAs are concentrated in the anticodon loop and core region, and play important roles in translation processes and stabilization of the tertiary structure of the tRNA molecules (57). Nevertheless, only three tRNA modifications have been reported to be responsible for growth ability under high temperature conditions, that were m^1^A58, m^5^s^2^U54 and *N*^7^-methylguanosine46 (m^7^G46) modifications of tRNA in *T. thermophilus* (58–61). In this study, we identified five genes responsible for six types of tRNA modifications, G^+^15, m^1^I57, m^1^A58, ac^4^C, m^2^G10 and m^2,2^G10 in the isolated thermosensitive mutants of *T. kodakarensis*. G^+^15, m^1^I57 and m^2,2^G10 are unique modifications found in many species of archaeal tRNAs (62–64). The association of these modifications, except for m^1^A58, with cellular thermotolerance of (hyper)thermophiles has not been reported so far.

Two of the identified genes *tk0760* (*tgtA*) and *tk1198* (*queE*) were related to archaeosine biosynthesis, and their actual participation in tRNA modification was confirmed by the disappearance of G^+^ in total tRNA nucleosides from FFH05 and FFH18 mutants. In addition, a partially thermosensitive mutant FFH14 had a transposon insertion at *tk2157*, located upstream of *arcS* (*tk2156*) (Supplementary Table S3), of which product (archaeosine synthase) catalyzes conversion of preQ_0_15-tRNA to G^+^15-tRNA as the final step in the G^+^ biosynthesis pathway in Euryarchaeota (44,65). The previous quantum mechanics calculations has showed that the G15-C48 Levitt base pair, a crucial tertiary interaction in the tRNA core, is stabilized by Mg^2+^, while the positively charged formamidine group at C_7_ of G^+^15 can stabilize the interaction with C48 as a mimic of the hydrated Mg^2+^ ion (66). Supporting this, we here confirmed ubiquitous presence of G^+^15 in total species of tRNAs in *T. kodakarensis* (Supplementary Table S4A) and observed lower *T*_m_ values of the total tRNAs lacking G^+^15 by 1.5–2°C (Table 2). The present results strongly suggested that thermal stabilization of tRNAs by the G^+^15 modification is one of factors important for the high cellular thermotolerance of *T. kodakarensis*.

TK0754, identified as a protein responsible for the thermosensitivity of FFH32, was estimated to form an ac^4^C modification based on the homology to TmcA from *E. coli* (48). It is known that ac^4^C modification locates at position 34 of bacterial and halophilic archaeal tRNAs (48, 62, 63). The C3′-*endo* ribose puckering of ac^4^C34 in bacterial tRNA^Met^ formed by TmcA or TmcAL confers stable codon–anticodon pairing at the wobble position, and thereby contributes to accurate decoding of the AUG codon by preventing misreading of the near-cognate AUA codon (48,67). LC–MS/MS analysis of tRNA nucleosides from FFH32 showed disappearance of ac^4^C, indicating that TK0754 was truly an archaeal ortholog of TmcA and a unique enzyme for the ac^4^C modification in *T. kodakarensis*. The roles of ac^4^C and *N*^4^-acetyl-2′-*O*-methylcytidine (ac^4^Cm) modifications in tRNA stabilization have been proposed based on the increase of these modifications in tRNAs from *P. furiosus* at elevated temperatures (68). However, the *T*_m_ of total tRNAs from FFH32 was only slightly lower than that of the control (Table 2). It was not clear whether this was because of a limited effect of the ac^4^C modification on tRNA thermostability or whether there were only a small amount of ac^4^C-modified tRNAs. Given a function of ac^4^C in accurate translation in bacteria, the frequency of translation errors under the high temperature conditions might increase in the *tmcA* mutant FFH32, consequently leading to the thermosensitive phenotype.

aTrm11 from *T. kodakarensis* (TK0981) and *P. abyssi* (PAB1283) showed *in vitro* activity to form m^2^G10 and m^2,2^G10 modifications (49,50,69). It has been reported that the m^2,2^G10 modification eliminates a hydrogen-bond donor in the Watson-Crick edge, avoiding formation of alternative structures in archaeal tRNAs (49). Increased levels of m^2^G, m^2,2^G and *N*^2^,*N*^2^,2′-*O*-trimethylguanosine (m^2,2^Gm) modifications were observed in tRNAs from *P. furiosus* at higher cultivation temperature (68). In this study, we confirmed that the relative abundance of m^2,2^G and melting property of the total tRNAs were not significantly changed by the *trm11*::Tn mutation in FFH35 (Figure 4 and Table 2). The shotgun analysis revealed that m^2^G10/m^2,2^G10 was present in a subset of tRNAs (Supplementary Table S4C) and the fragments bearing m^2^G10/m^2,2^G10 were absent in FFH35 (Supplementary Figure S8). The severe growth phenotype of FFH35 at the supraoptimal temperature suggested importance of the m^2^G10/m^2,2^G10-modified tRNA species in thermotolerance of *T. kodakarensis*.

TK1328, an archaeal ortholog of TrmI (aTrmI) forming m^1^A at positions 58 and 57 *in vitro* (51–53), was also identified as a gene responsible for the thermosensitivity of *T. kodakarensis* mutant FFH20 (*trmI*::Tn). In *T. thermophilus*, the importance of m^1^A58 in cellular thermotolerance has been demonstrated by growth defect of the *trmI*-disrupted strain at 80°C (58). After this observation, Shigi *et al.* found that m^1^A58 was required for efficient 2-thiouridylation of m^5^U54 (55). The resulting m^5^s^2^U54 with C3′-*endo* conformation was proposed to stabilize the interaction between the D and T loops, leading to stabilization of tRNA tertiary structure (70) and consequent thermotolerance for *T. thermophilus* cells (59,60). In tRNAs from FFH20, we detected hypomodification of m^5^s^2^U54 in addition to the absence of m^1^I57/m^1^A58 (Figure 4). These results agreed with the common machinery for the m^1^A58-induced formation of m^5^s^2^U54 between the bacterium *T. thermophilus* and archaeon *T. kodakarensis*. The absence of m^1^I was also detected in tRNAs from the mutant, which was consistent with the *in vitro* activity of aTrmI from *P. abyssi* for formation of m^1^A57 (51) and conversion of m^1^A57 to m^1^I57 by using extracts of *Haloferax volcanii* and *P. furiosus* (71,72). The remaining m^1^A detected in tRNAs from FFH20 was probably m^1^A9 produced by another tRNA methyltransferase Trm10 (TK0422) (54). We further observed a decrease in *T*_m_ by 2°C for tRNAs from FFH20 as observed for the *trmI*-deleted mutant of *T. thermophilus* (55). The results supported the proposed role of m^5^s^2^U in thermal stabilization of the tRNA core structure also in hyperthermophilic archaea.

tRNA modifications serve functions in not only stabilization of tRNA tertiary structure and maintenance of translational fidelity, but also discrimination and turnover of tRNA molecules. In *Saccharomyces cerevisiae*, it has been demonstrated that one or more mutations in the subsets of tRNA modification genes can result in negative phenotypes such as temperature-sensitive growth caused by degradation of mature tRNAs not bearing the modifications (73). This pathway for tRNA turnover is now known as rapid tRNA decay (RTD). Hyperthermophilic archaea located at the nearest branches in the phylogenetic tree often possess similar but simple machineries for DNA replication, transcription and translation to those in Eucarya. It was assumed that unknown tRNA turnover linked with modifications might be functional in hyperthermophilic archaea as a prototype of eukaryotic RTD and relate to the thermosensitivity of the mutants isolated in this study.

This study also identified two genes *tk1803* (putative dipeptide/oligopeptide ABC transporter permease) and *tk2145* (hypothetical protein) related to the high thermotolerance of *T. kodakarensis*. The identification of the transporter gene *tk1803* suggested that the cells may require uptake of some compounds easily degraded at extremely high temperatures. The growth defect at 93°C caused by transposon insertions within *tk0672* (putative glutamate synthase β-subunit), *tk1402* (polysaccharide deacetylase) and *tk0647* (hypothetical protein) were unable to be restored by introduction of the corresponding intact gene. This phenomenon may be because of insufficient function of the intact gene in the shuttle vector owing to weak activity of the *pyrF* promoter, or polar effects causing reduced expression of genes close to the transposon insertion. The use of a strong promoter or introduction of neighboring genes around the mutation site would help clarify these results. The function of these identified genes other than tRNA modification genes are also quite interesting. Further studies are expected to elucidate the role of these in cellular thermotolerance. On the other hand, this study did not identify the genes reported previously to be involved in hyperthermotolerance of *T. kodakarensis* other than reverse gyrase gene, such as genes for chaperonin CpkB (3) and polyamine biosynthesis (10). Considering the 2306 genes estimated in the *T. kodakarensis* genome, the present screening of 1957 clones in the random mutant library was not sufficient for comprehensive isolation of the target mutants despite the enough coverage by the transposon-inserted DNA library. Screening of more mutant clones will identify additional genes related to the cellular thermotolerance. Moreover, the random mutant library can be also applied for isolation of various kinds of mutants depending on the selection strategy. It is expected that isolation and analyses of mutants screened from the library will help us to identify and elucidate novel functions in hyperthermophilic archaea.

## Supplementary Material

Supplementary Data
