# Supplementary material for: Random mutagenesis of a hyperthermophilic archaeon identified tRNA modifications associated with cellular hyperthermotolerance
Source: Nucleic Acids Res. 2019 Jan 3;47(4):1964–76. doi: 10.1093/nar/gky1313 (PMC6393311; doi:10.1093/nar/gky1313)
Supplement: Supplementary Data [file gky1313_supplemental_files.pdf]

SUPPLEMENTARY MATERIAL:

Random mutagenesis of a hyperthermophilic archaeon identified tRNA modifications associated with cellular hyperthermotolerance

Izumi Orita[1)], Ryohei Futatsuishi[1)], Kyoko Adachi[1)], Takayuki Ohira[2)], Akira Kaneko[1)], Keiichi Minowa[2)], Miho Suzuki[1)], Takeshi Tamura[1)], Satoshi Nakamura[1)], Tadayuki Imanaka[3)], Tsutomu Suzuki[2)], and Toshiaki Fukui[1)*]

School of Life Science and Technology, Tokyo Institute of Technology, Yokohama, Japan[1)], Department of Chemistry and Biotechnology, Graduate School of Engineering, University of Tokyo, Tokyo, Japan[2)], The Research Organization of Science & Technology, Ritsumeikan University, Kusatsu, Japan[3)].

Content:

Supplementary Results

Table S1, S2, S3, S4

Figure S1, S2, S3, S4, S5, S6, S7, S8, S9

Supplementary Results

Isolation of 6-methylpurine-resistant mutant for validation of the random mutant library of *T. kodakarensis*

6-Methylpurine (6MP) is known to be toxic for hypoxanthine/guanine phosphoribosyltransferase-positive cells, and TK0664 corresponds to this enzyme in *T. kodakarensis* (1). Three 6MP-resistant mutants could be obtained from the mutant library on 6MP-containing plate medium and showed normal growth on ASW-YT-S^0 medium containing 0.5 mM 6MP at 85˚C (Fig. S1 (B)). As expected, sequencing analysis of the rescued plasmids clarified that all the three mutants had transposon insertion within *tk0664* at which direct repeat region was 139-147 bp (Fig. S1 (C)).

Disruption of predicted restriction endonuclease gene.

The whole genome analysis of *T. kodakarensis* identified 9 genes encoding probable restriction endonucleases. Among them, *tk2239* was disrupted in the Δ*pdaD* strain aiming at improving transformation efficiency, because TK2239 shared 28% identity with a type II restriction endonuclease PhoI derived from the closely related hyperthermophile *Pyrococcus horikoshii*.
A DNA fragment of *tk2239* together with the 1-kbp flanking regions was amplified with gDNA of *T. kodakarensis* as a template and a primer set of tk2239_up/tk2239_down. The amplified fragment was inserted into pUD3 harboring *pyrF* marker (2) at the HincII site. Inverse PCR with a primer pair tk2239_Inv1/tk2239_Inv2 was then carried out to amplify the flanking regions along with the plasmid backbone, thereby removing the coding region. The amplified fragment was self-ligated after 5'-phosphorylation, and the resulting plasmid was designed as pUD3-Δ2239. *T. kodakarensis* strains Δ*pdaD* (3) and KU216 (4) were transformed with pUD3-Δ2239 to obtain strains of Δ2239 (Δ*pdaD* Δ*pyrF* Δ*tk2239*) and KD2239 (Δ*pyrF* Δ*tk2239*), respectively. Selection of uracil-prototrophic transformants and 5-fluoroorotic acid (5-FOA)-resistant uracil-autotrophic transformants formed by pop-in and pop-out recombination, respectively, were performed according to the procedure described previously (5).

Upon transformation with the transposon-inserted library DNA, the disruptant Δ2239 showed maximum transformation efficiency of 6 x10^3 CFU/μg DNA, that was higher than 3 x10^3 CFU/μg DNA of the parent strain. Further mutagenesis experiments were thus carried out by using Δ2239 as a host strain. The strain KD2239 was used as a control strain in growth measurement of the isolated mutant strains.

REFERENCES

1. Hileman, T.H. and Santangelo, T.J. (2012) Genetics Techniques for *Thermococcus kodakarensis*. *Front Microbiol*, **3**, 195.

2. Kobori, H., Ogino, M., Orita, I., Nakamura, S., Imanaka, T. and Fukui, T. (2010) Characterization of NADH oxidase/NADPH polysulfide oxidoreductase and its unexpected participation in oxygen sensitivity in an anaerobic hyperthermophilic archaeon. *J Bacteriol*, **192**, 5192-5202.

3. Fukuda, W., Morimoto, N., Imanaka, T. and Fujiwara, S. (2008) Agmatine is essential for the cell growth of *Thermococcus kodakaraensis*. *FEMS Microbiol Lett*, **287**, 113-120.

4. Sato, T., Fukui, T., Atomi, H. and Imanaka, T. (2003) Targeted gene disruption by homologous recombination in the hyperthermophilic archaeon *Thermococcus kodakaraensis* KOD1. *J Bacteriol*, **185**, 210-220.

5. Sato, T., Fukui, T., Atomi, H. and Imanaka, T. (2005) Improved and versatile transformation system allowing multiple genetic manipulations of the hyperthermophilic archaeon *Thermococcus kodakaraensis*. *Appl Environ Microbiol*, **71**, 3889-3899.

6. Atomi, H., Fukui, T., Kanai, T., Morikawa, M. and Imanaka, T. (2004) Description of *Thermococcus kodakaraensis* sp. nov., a well studied hyperthermophilic archaeon previously reported as *Pyrococcus* sp. KOD1. *Archaea*, **1**, 263-267.

7. Fukuda, W., Morimoto, N., Imanaka, T. and Fujiwara, S. (2008) Agmatine is essential for the cell growth of *Thermococcus kodakaraensis*. *FEMS Microbiol Lett*, **287**, 113-120.

8. Santangelo, T.J., Cubonova, L. and Reeve, J.N. (2008) Shuttle vector expression in *Thermococcus kodakaraensis*: contributions of cis elements to protein synthesis in a hyperthermophilic archaeon. *Appl Environ Microbiol*, **74**, 3099-3104.

Table S1. Strains and plasmids used in this study

Strains	Genotypes	Source or reference
Escherichia coli		
DH5α	φ80*lacZ*ΔM15 *recA1 endA1 gyrA96 thi*-1 *hsdR17*($r_k^- m_k^+$) *supE44 relA1 deoR* Δ(*lacZYA-argF*) U169 *phoA*	
HST08	F^- φ80*lacZ*ΔM15 *recA1 endA1 supE44 thi*-1 *relA1* Δ(*lacZYA-argF*) U169 Δ(*mrr-hsdRMS-mcrBC*) *gyrA96 phoA* Δ*mcrA* λ^-	Takara Bio
EC100D *pir*-116	F^- *mcrA recA1 endA1* Δ(*mrr-hsdRMS-mcrBC*) φ80*lacZ*ΔM15 Δ*lacX74 araD139* Δ(*ara, leu*)*7697 galU galK* λ^- *rpsL* (*Str*^{R}) *nupG pir-116*(*DHFR*)	Epicentre
Thermococcus kodakarensis		
KOD1	Wild type	(6)
KU216	Δ*pyrF* (uracil auxotroph)	(4)
Δ*pdaD*	Δ*pdaD* Δ*pyrF* (agmatine and uracil auxotroph)	(7)
Δ2239	Δ*pdaD* derivative, Δ*tk2239*	This study
KD2239	KU216 derivative, Δ*tk2239*	This study
Plasmids		
pUC118	Cloning vector, Amp^r	Takara Bio
pUD3	pUC118 derivative, *pyrF* marker cassette	(2)
pUD3-Δtk2239	pUD3 derivative, *tk2239 del**	This study
pMOD3	ColE1 ori, Amp^r, R6Kγ ori, mosaic end sequences, MCS	Epicentre
pMOD3-pdaD-Gm	pMOD3 derivative, *pdaD*$_{Tk}$, Gm^r	This study
pLC71	*E. coli-T. kodakarensis* shuttle vector, Amp^r, Km^r, *trpE*$_{Tk}$, *hmg*$_{Pf}$	(8)
pLCS	pLC71 derivative, Δ*trpE*$_{Tk}$::MCS (NdeI-StuI-SpeI-NheI-SphI)	This study

* Indicates an insert used for targeted gene deletion.

Table S2. Primer sequences used in this study.

Primer	Sequence（5'→3'）	Site and others
tk2239_up	CTTGCTCCCGCTCATCTTGCCCTTC	
tk2239_down	GAAGCTCTTCATAGCAAGGCTGTGCGAG	
tk2239_Inv1	TTCGATCACCGTTTTATTTTTCTGTCG	
tk2239_Inv2	GCAAGGTATATAACCCTCCTCACGC	
tk0149-f	GTTCTAGAGGCCTTCCTTGACCTTGTA	
tk0149-r	AATCTAGATCAGTAGGGGAACATGACGACG	
tk0149-N	ATGAGCTGGACAACCCCAAAG	
GAT-f2	TGCCGGGTGACGCACACCGT	
GAT-r2-Age	GCATACCGGTACAAGGACAATTAACAGTT	AgeI
pMOD3-MCS-Fw	ATTCAGGCTGCGCAACTGT	
pMOD3-MCS-Rv	GTCAGTGAGCGAGGAAGCGGAAG	
pMOD3-SqFp	GCCAACGACTACGCACTAGCCAAC	
pMOD3-SqRp	GAGCCAATATGCGAGAACACCCGAGAA	
pLC71-inv1DKanr	TACTAGTGCTAGCGCATGCCTTCTATCGCCTTCTTGACGAGTTCTTCTG	SpeI-NheI-SphI
pLC71-inv2	GGCCTCATATGCATCACCTTTTTAACGGCCCTCTC	NdeI
mEzTN5SqFp	AAACATGAGAGCTTAGTACGTACTATC	
mEzTN5SqRp	TTTTTATTTGTTAACTGTTAATTGTCCT	
EzTN5SqFp	GGTTGAACTGCCAACGACTACGCACTA	
EzTN5SqRp	CGAATTCGAGCCAATATGCGAGAACACCCGAGAA	
tk0754-Fw-NdeI	GGAATTCCATATGACCGTCAAGGTCCGCTTT	NdeI (FFH02)
tk0754-Rv-SphI	ACATGCATGCTCAGGTGAGAAGCCTCTTGTA	SphI (FFH02)
tk0760_NdeN	GGAATTCCATATGGTCGATTTCAGGTTTGAGGTC	NdeI (FFH18)
tk0760_XbaC	GCTCTAGACTACTTCTCGACTCCCCTCCTAAC	XbaI (FFH18)
tk0981_NdeN	GGAATTCCATATGCTCTACGTGGAGATACTCGGAAAC	NdeI (FFH35)
tk0981_SpeC	CTGTTATAGTTACTAGTTCATTTCTCAAACACATAGAAGTACC	SpeI (FFH35)
tk1198-Fw-NdeI	GGAATTCCATATGAGGGTGATAATGGCCGA	NdeI (FFH05)
tk1198-Rv-SphI	ACATGCATGCTCAAAGCACGTTCAGGTA	SphI (FFH05)
Tk1328_NdeN	GGAATTCCATATGATATCAGTCGGAGATAGAGTTCTTCTC	NdeI (FFH20)
Tk1328_XbaC	GCTCTAGAGTCACAGCTTCCTGAGGAAGGTTATG	XbaI (FFH20)
tk0672-Fw-NdeI	GGAATTCCATATGAGGTTCTACATCTGCAGGGAA	NdeI (FFH15)
tk0672-Rv-SphI	ACATGCATGCTCAGCACTTCCCGTTCGC	SphI (FFH15)
tk1803-Fw-NdeI	GGATTCCATATGGGATTTGGTAGGTACGTGCTGAT	NdeI (FFH12)
tk1803-Rv-SpeI	GGACTAGTTCACACATTCTGGGATGCACCAACC	SpeI (FFH12)
tk0647-Fw-NdeI	GGAATTCCATATGAACATCCAGAATGCTG	NdeI (FFH27)
tk0647-Rv-SpeI	GGACTAGTTTAATCAACTTTGAAACACAGAACA	SpeI (FFH27)
tk1402-Fw-NdeI	GGAATTCCATATGAAAAAATTGCTGGTAATAA	NdeI (FFH08)
tk1402-Rv-SphI	ACATGCATGCTCAACGTTCGTGGTTGGGAGA	SphI (FFH08)
tk2145-Fw-NdeI	GGAATTCCATATGGCTGAAAGCATTGGCG	NdeI (FFH22)
tk2145-Rv-SpeI	GGACTAGTCACTCCTTGTTCAGCCCG	SpeI (FFH22)

Table S3. Transposon insertion sites in *T. kodakarensis* partially thermosensitive mutants isolated by random mutagenesis.

Predicted function	Transposon insertion site	Annotation	Mutant strain (Direct repeat region [bp no.])
metabolism	*tk0260*	aromatic aminotransferase	FFH09 (308-316)
	tk1405	phosphoenolpyruvate carboxykinase	FFH13 (849-857)
	tk1406	maltodextrin phosphorylase	FFH19 (863-871 and 430-bp deletion)
	tk0262	chorismate synthase	FFH37 (691-699)
	tk0245	imidazoleglycerol-phosphate dehydratase HisB	FFH38 (311-319)
cellular process	*tk0635*	chemotaxis histidine kinase, frame shift	FFH01 (134-142)
	tk1179	adenylate cyclase, class 2	FFH28 (101-109)
	tk1361	MCM2/3/5 family DNA replication licensing factor	FFH29 (420-428)
	tk0701	ATPase (MinD superfamily P-loop ATPase containing an inserted ferredoxin domain)	FFH31 (470-478)
transport	*tk0658*	simple sugar transport system ATP-binding protein	FFH06 (1076-1084)
	tk0707	ABC-type iron(III)-siderophore transport system permease	FFH10 (not determined)[1)]
	tk1771	ABC-type maltodextrin transport system maltodextrin-binding periplasmic protein	FFH23 (1259-1267)
degradation	*tk0699*	zinc-dependent protease	FFH26 (289-297)
	tk1169	exosome complex RNA-binding protein Csl4	FFH34 (246-254)
	tk2252	proteasome-activating nucleotidase	FFH03 (76-84)<br>FFH04 (547-555)
mobile genetic element	*tk0575*	integrase, *N*-fragment	FFH39 (25-33)
unknown	*tk0437*	hypothetical protein	FFH07 (1210-1218)
	tk2157	hypothetical protein	FFH14 (4-12)
	tk2174	hypothetical protein	FFH30 (127-135)

1) Sequencing of the junction regions was unsuccessful.

Table S4A. List of predicted RNA fragments with G$^+$15 (RNase T_1-digestion)

No.	Sequence of RNA fragments	Molecular mass	*m/z* (-2)	Mass spectrum	CID	Possible tRNAs	# of tRNAs
1	UCUAG$^+$ACUGp	2935.40	1466.69	Observed	Not assigned	Val(CAC), Val(GAC)	2
2	CCUAG$^+$CCAGp	2933.43	1465.71	Observed	Assigned	Arg(CCT)	1
3	CCUAG$^+$UCUGp/ UCUAG+CCUGp/ CUUAG+CCUGp	2911.38	1454.68	Observed	Not assigned	Arg(CCG), Arg(GCG), Arg(TCG), Gly(CCC), Gly(GCC), Gly(TCC), Lys(CTT), eMet(CAT)	8
4	CUCAG$^+$CCUGp/ CCUAG+CCUGp/ CCUAG+CUCGp	2910.40	1454.19	Observed	Not assigned	Ala(CGC), Ala(GGC), Ala(TGC), Ile(GAT), Ile(CAT), Lys(TTT), Phe(GAA), Ser(CGA), Ser(GCT), Ser(GGA), Ser(TGA), Thr(TGT), Tyr(GTA), Thr(GGT), Thr(CGT)	15
5	UCUAG$^+$AUGp	2630.35	1314.17	Observed	Assigned	Val(TAC)	1
6	CCUAG$^+$CAGp	2628.39	1313.19	Observed	Assigned	Arg(TCT)	1
7	CUUAG$^+$UUGp	2607.33	1302.66	Observed	Assigned	Asn(GTT)	1
8	CCUAG$^+$AGp	2323.34	1160.66	Observed	Not assigned	Cys(GCA)	1
9	CAG$^+$CUAGp	2323.34	1160.66	Observed	Assigned	iMet(CAT)	1
10	UAG$^+$CUUGp	2301.30	1149.64	Observed	Assigned	Pro(CGG), Pro(GGG), Pro(TGG)	3
11	UAG$^+$CCUGp	2300.32	1149.15	Observed	Assigned	His(GTG), Trp(CCA)	2
12	UAG$^+$CCCGp	2299.33	1148.66	Observed	Assigned	Asp(GCT), Glu(CTC), Glu(TTC)	3
13	AG$^+$CCUGp	1994.29	996.14	Observed	Assigned	Leu(CAA), Leu(CAG), Leu(GAG), Leu(TAA), Leu(TAG)	5
14	UAG$^+$CG	1689.25	843.62	Observed	Assigned	Gln(CTG), Gln(TTG)	2

Table S4B. List of RNA fragments with m^{1}A58 (RNase A-digestion)

No.	Sequence of RNA fragments	Molecular mass	*m/z* (-2)	Mass spectrum	CID	Possible tRNAs	# of tRNAs
1	Cmm^{1}Im^{1}AAUp	1659.27	828.63	Observed	Assigned	Ala(CGC), Ala(GGC), Arg(GCG), Arg(TCG), Asp(GTC), Cys(GCA), Glu(CTC), Glu(TTC), Gly(CCC), Gly(GCC), Gly(TCC), His(GTG), iMet(CAT), Leu(CAA), Leu(CAG), Leu(GAG), Leu(TAG), Phe(GAA), Pro(CGG), Pro(GGG), Pro(TGG), Ser(CGA), Ser(GCT), Ser(GGA), Ser(TGA), Thr(CGT), Thr(GGT), Thr(TGT), Trp(CCA), Tyr(GTA)	30
2	CmGm^{1}AAUp	1660.26	829.12	Observed	Assigned	Ala(TGC), Arg(CCG), Arg(TCT), Asn(GTT), Gln(CTG), Gln(TTG), Leu(TAA), Lys(CTT), Lys(TTT), Val(CAC), Val(GAC), Val(TAC)	12
3	Cmm^{1}Im^{1}AAGCp	2003.33	1000.66	Observed	Assigned	Ile(CAT), eMet(CAT)	2
4	CmGm^{1}AAGCp	2004.32	1001.15	Observed	Assigned	Arg(CCT), Ile(GAT)	2

Table S4C. List of RNA fragments with m^{2}G10/m^{2,2}G10 or m^{2,2}G26 (RNase T_1-digestion)

No.	Sequence of RNA fragments	Molecular mass	*m/z* (-1 or -2)	Mass spectrum	CID	Possible tRNAs	# of tRNAs
1	Um^{1}Am^{2}Gp (position 10)	1008.16	1007.15	Observed	Assigned	Ala(CGC), Ala(GGC), Ala(UCG), Arg(CCT), Arg(TCT), Asn(GTT), Gly(CCC), Gly(GCC), Gly(TCC), Leu(CAG), Leu(TAG), Lys(CTT), Lys(TTT) , Ile(CAT), eMet(CAT), Phe(GAA), Ser(CGA), Ser(GCT), Ser(GGA), Thr(CGT), Thr(TGT), Tyr(GTA)	22
2	m^{2,2}GUGp (position 10)	1024.15	1023.14	Observed	Assigned	Asp(GTC), Gln(CTG), Gln(TTG), Glu(CTC), Glu(TTC), His(GTG), Trp(CCA)	7
3	Cm^{2,2}GCCGp (position 26)	1633.25	815.62	Observed	Assigned	Ala(CGC), Ala(GGC), Arg(TCT), Asn(GTT), Phe(GAA), Ser(GCT)	6
4	Cm^{2,2}GUCGp (position 26)	1634.23	816.11	Observed	Assigned	Ala(TGC), Arg(CCG), Arg(TCG)	3

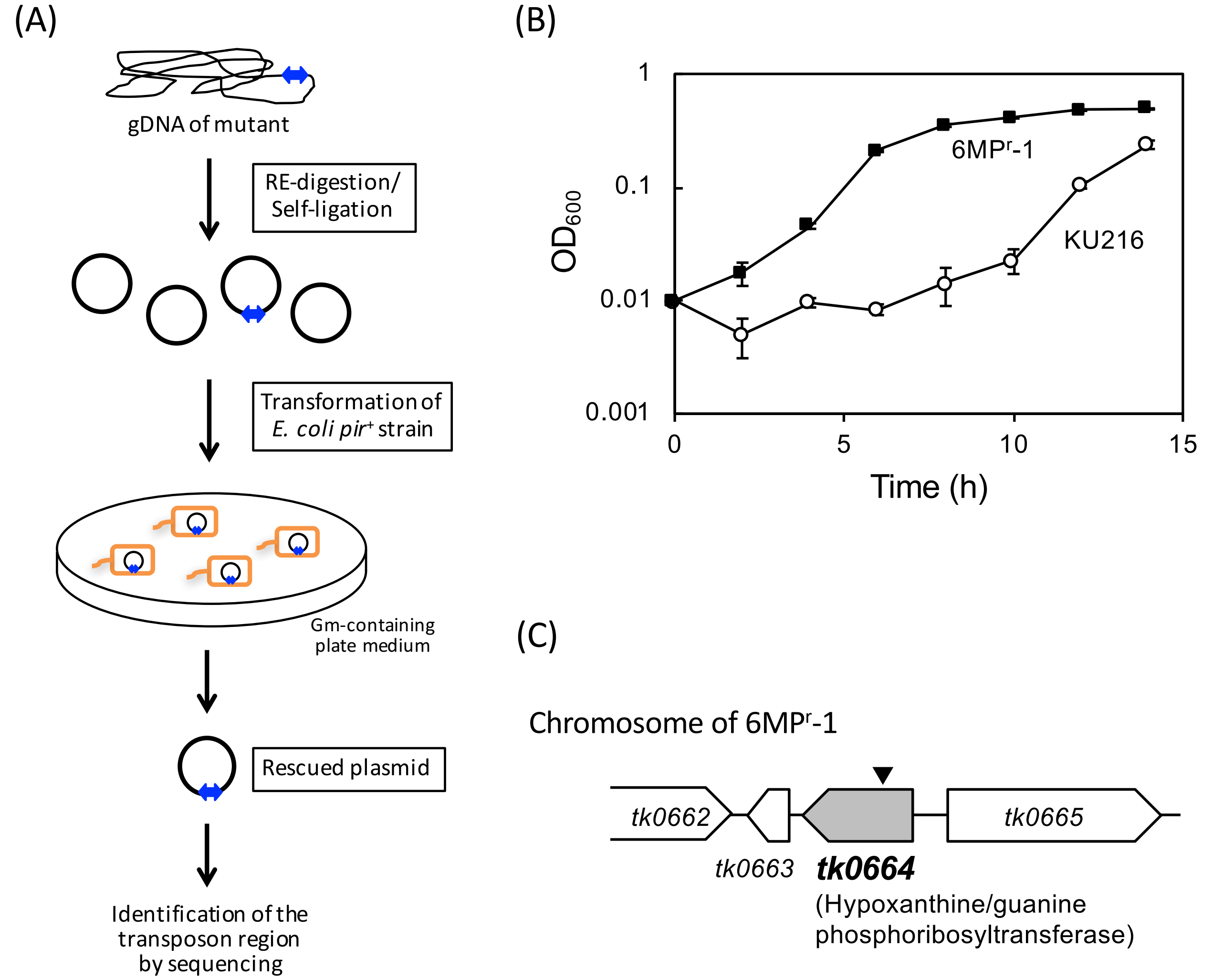

Fig. S1. (A) Scheme of plasmid rescue for identification of a transposon-inserted region in the isolated mutant. The blue double-headed arrow indicates the artificial transposon region containing R6Kg ori and gentamycin (Gm)-resistant cassette. (B) Growth of 6-methylpurine (6MP)-resistant mutant $6MP^r$-1 (■) and control strain KU216 (○) of *T. kodakarensis* in ASW-YT-S^0 medium containing 0.5 mM 6MP at 85˚C. (C) Gene organization of the transposon-inserted region in *T. kodakarensis* $6MP^r$-1 chromosome. The transposon region in $6MP^r$-1 was identified within *tk0664* with direct repeat of 148-156 bp region of the gene.

[tRNA modification mutants]

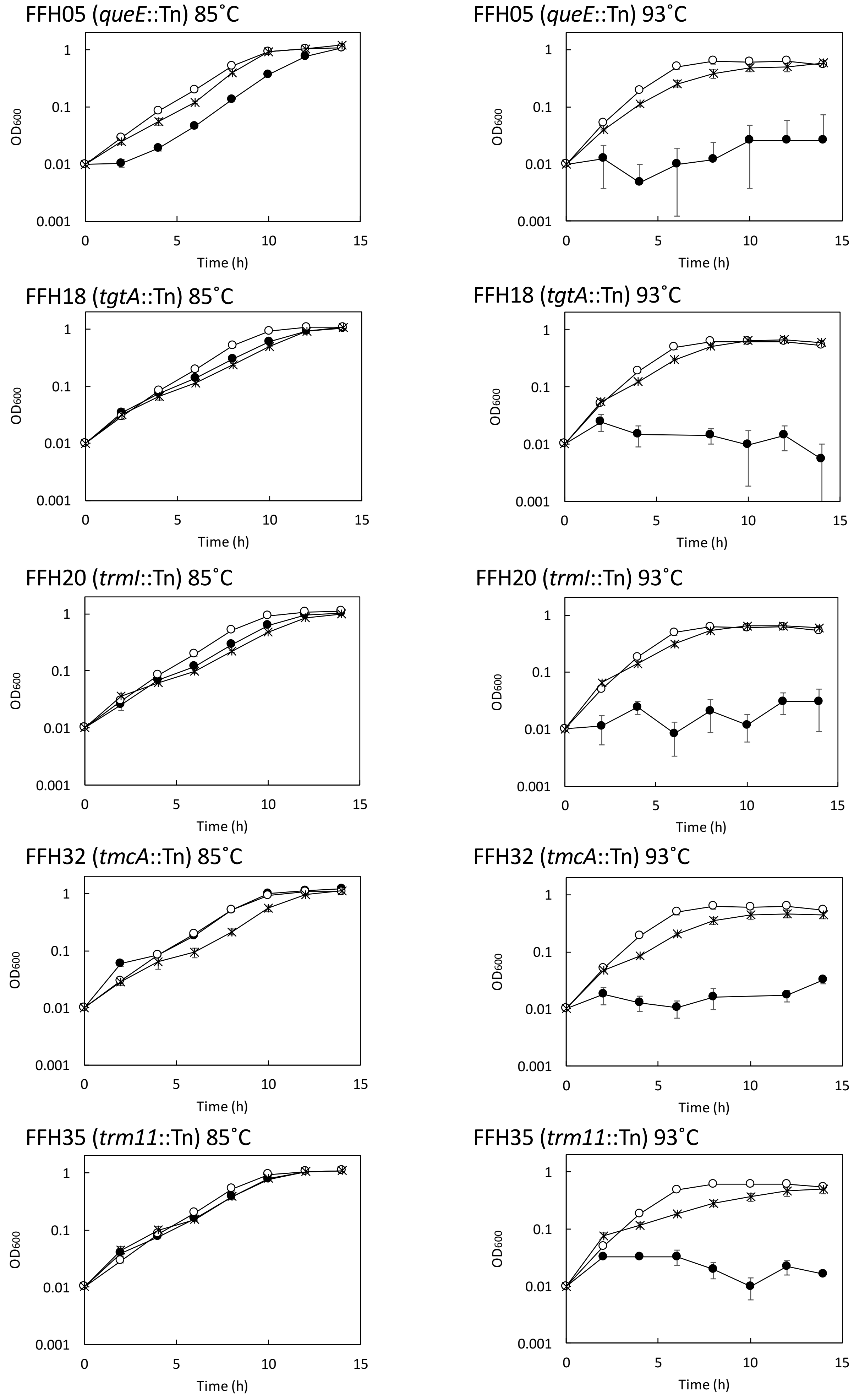

Fig. S2. Growth properties of *T. kodakarensis* tRNA modification mutants (●), their complemented strains (✕), and control strain KD2239 (○) (n = 3).

[Other mutants]

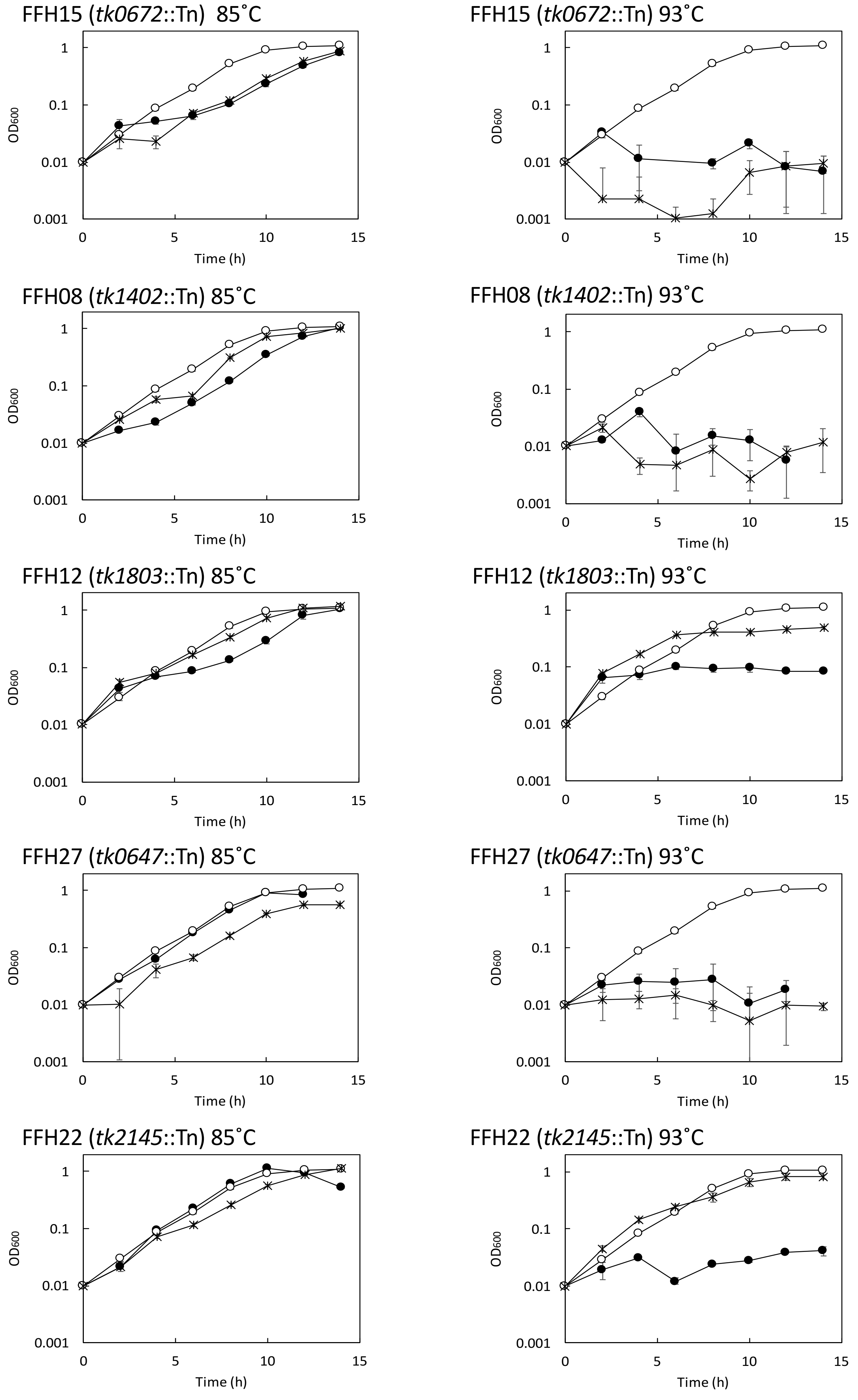

Fig. S3. Growth properties of *T. kodakarensis* thermosensitive mutants other than tRNA modification mutants (●), their complemented strains (✕), and control strain KD2239 (○) (n = 3).

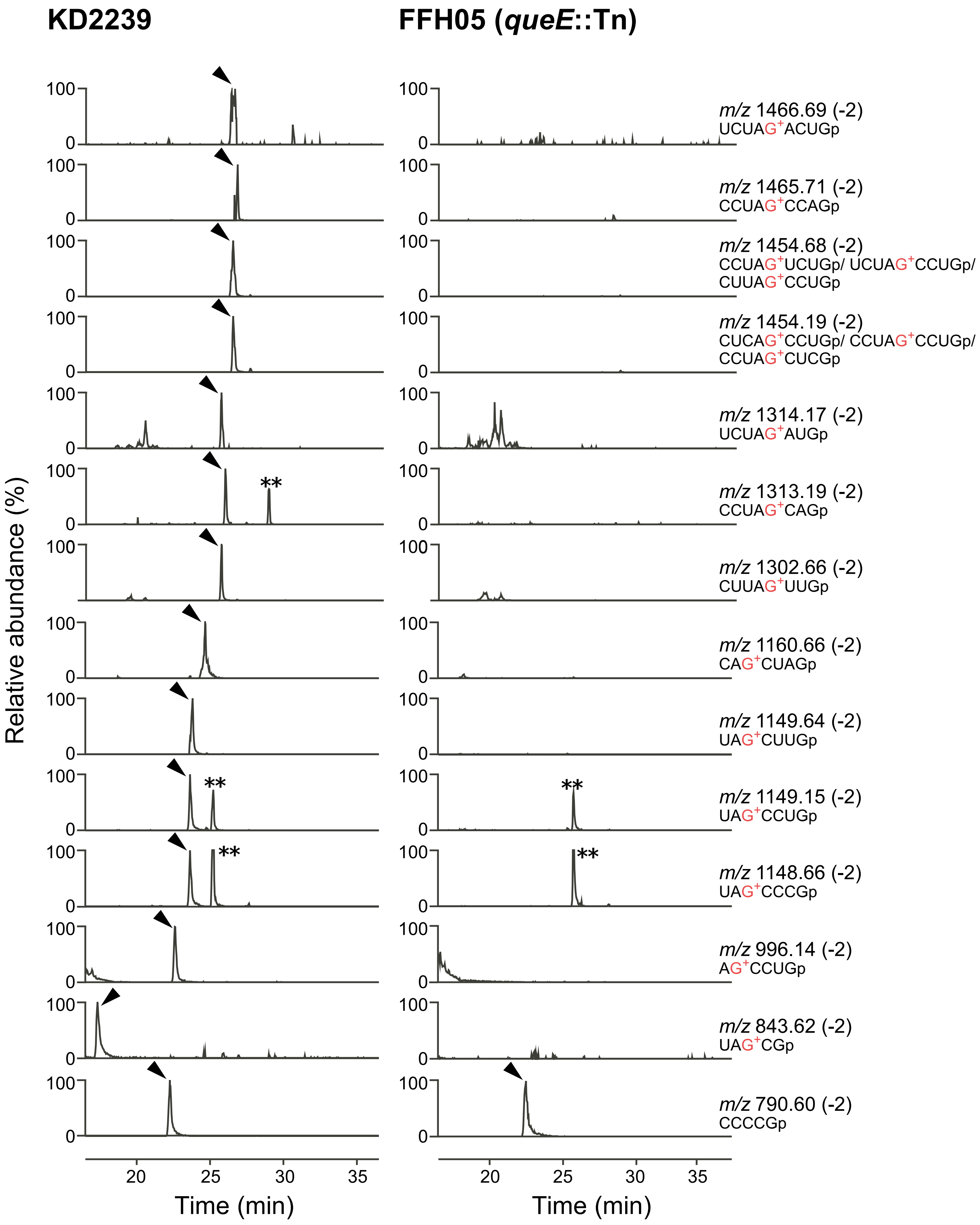

Fig. S4. Shotgun analysis of tRNA fragments generated by RNase T_1-digestion of tRNA extracted from *T. kodakarensis* KD2239 and FFH05 (*queE*::Tn). The panels represent the mass chromatograms that detect negatively charged ions of RNA fragment containing a nucleotide at position 15. The relative abundance represents relative intensity of each peak normalized by the intensity of CCCCGp fragment. The peaks of the corresponding fragments are indicated by black arrowheads. Double asterisks indicate unassigned ions.

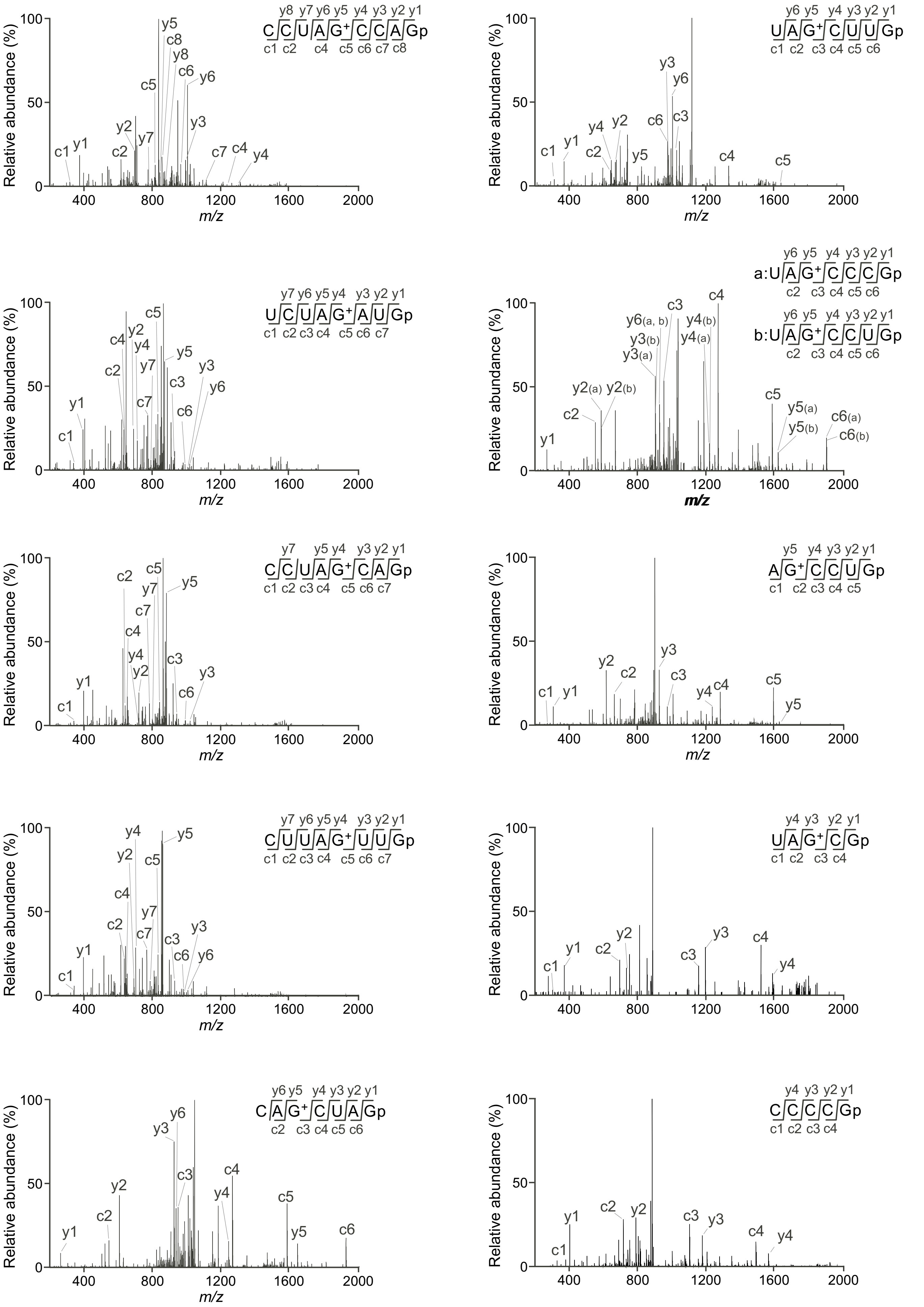

Fig. S5. CID spectra of ten RNA fragments having $G^{+}15$ along with CCCCGp fragment used as the internal standard in Fig. S4. Negatively charged ions of the individual fragments were used as precursor ions for CID analysis. The product ions in each the CID spectrum are assigned on the sequences.

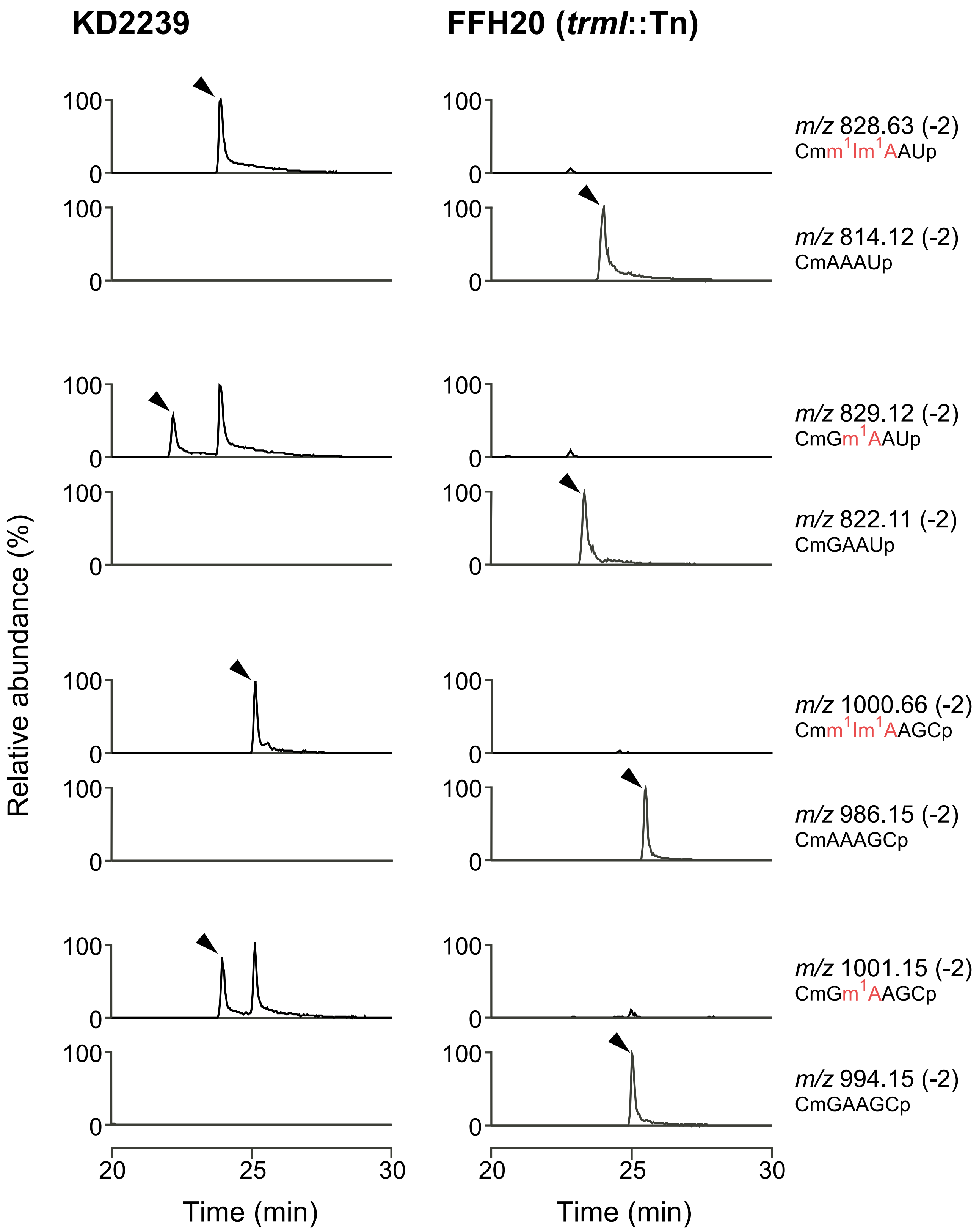

Fig. S6. Shotgun analysis of tRNA fragments generated by RNase A-digestion of tRNA extracted from *T. kodakarensis* KD2239 and FFH20 (*trmI*::Tn). The panels represent the mass chromatograms that detect divalent negatively charged ions of RNA fragment containing nucleotides from positions 56 to 60. The relative abundance represents relative intensity of each peak normalized by the highest peak. The peaks of the assigned fragments are indicated by black arrowheads

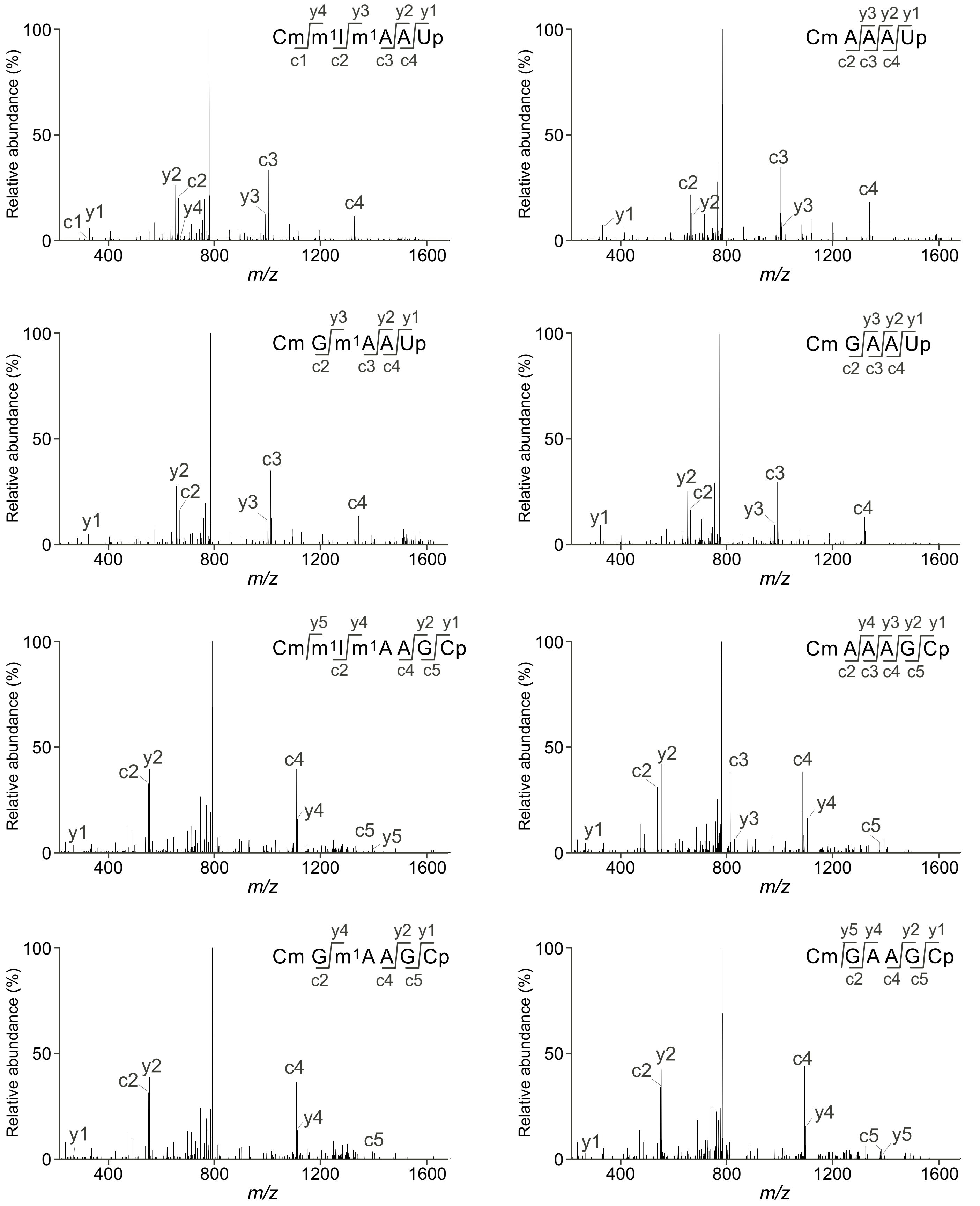

Fig. S7. CID spectra of eight RNase A digested-RNA fragments detected in Fig. S6. Negatively charged ions of the individual fragments were used as precursor ions for CID analysis. The product ions in each the CID spectra are assigned on the sequences.

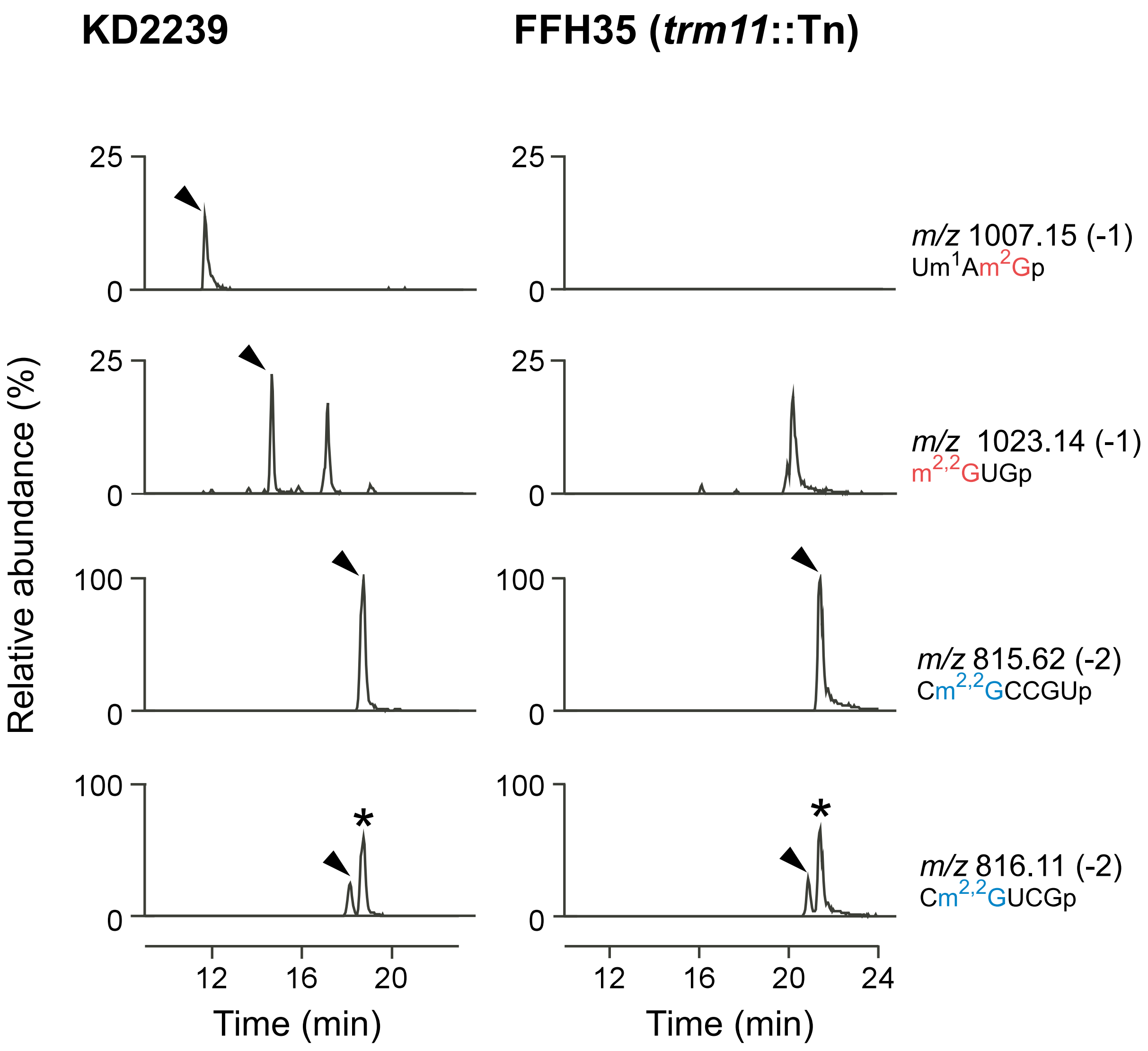

Fig. S8. Shotgun analysis of tRNA fragments generated by RNase T_1-digestion of tRNA extracted from *T. kodakarensis* KD2239 and FFH35 (*trm11*::Tn). The panels represent the mass chromatograms that detect monovalent or divalent negatively charged ions of RNA fragments containing the nucleotide at position 10 or 26. The relative abundance represents relative intensity of each peak normalized by intensities of $Cm^{2,2}GCCGp$ fragments. The peaks of the assigned fragments are indicated by black arrowheads. Asterisks indicate peaks of $Cm^{2,2}GCCGp$ fragments. Guanosines at positions 10 and 26 are shown in red and blue, respectively.

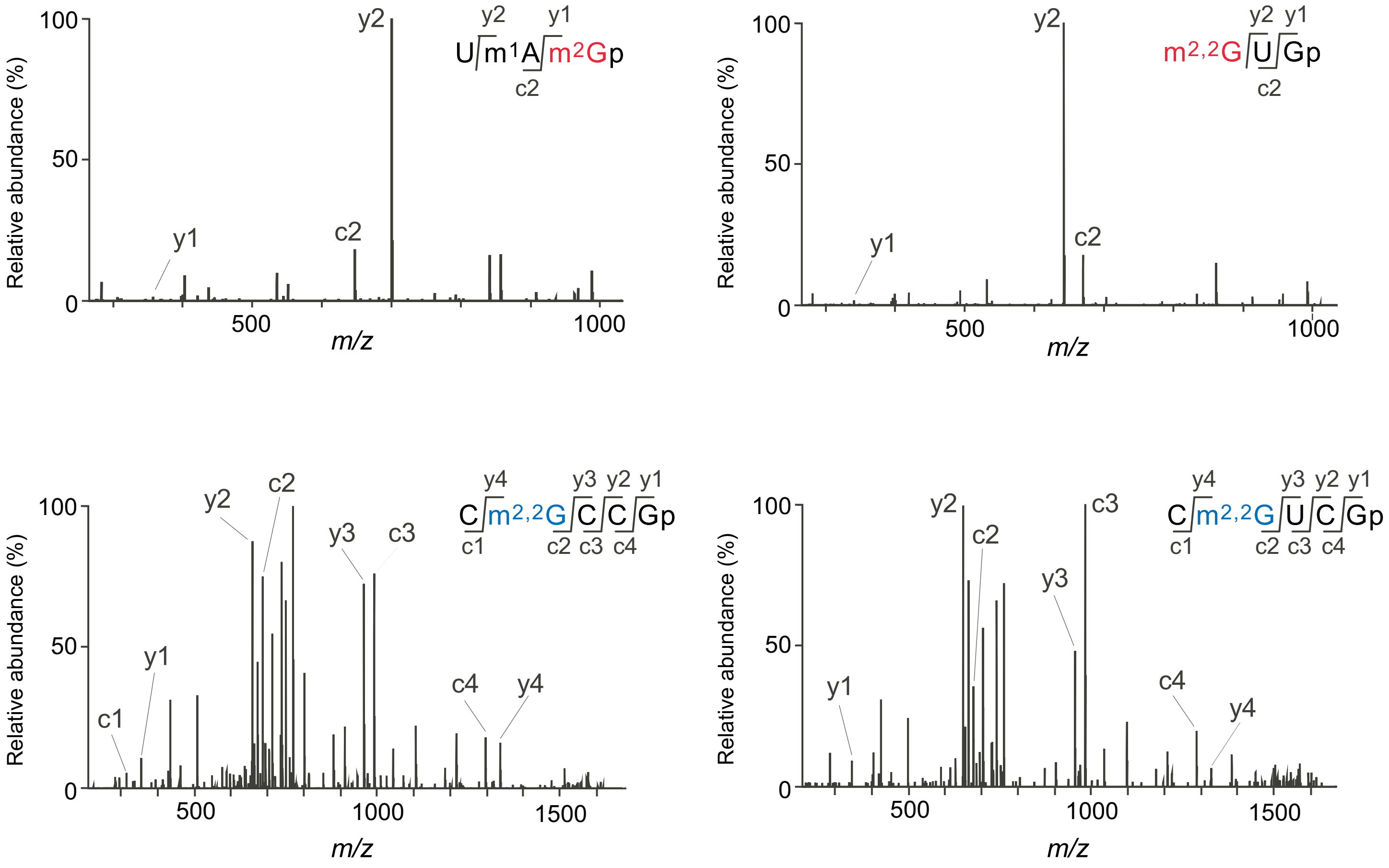

Fig. S9. CID spectra of four RNase T_1-digested tRNA fragments containing $m^2G/m^{2,2}G$ from *T. kodakarensis* KD2239 detected in Fig. S8. Monovalent or divalent negative ions were used as precursor ions for CID analysis. The product ions in each the CID spectra are assigned on the sequences. Guanosines at positions 10 and 26 are shown in red and blue, respectively.
